# Alzheimer’s Disease: Exploring the Landscape of Cognitive Decline

**DOI:** 10.1021/acschemneuro.4c00339

**Published:** 2024-10-11

**Authors:** Rumiana Tenchov, Janet M. Sasso, Qiongqiong Angela Zhou

**Affiliations:** CAS, a division of the American Chemical Society, Columbus Ohio 43210, United States

**Keywords:** Alzheimer’s disease, pathogenesis, aging, amyloid beta plaques, tau protein tangles, protein aggregation, biomarker

## Abstract

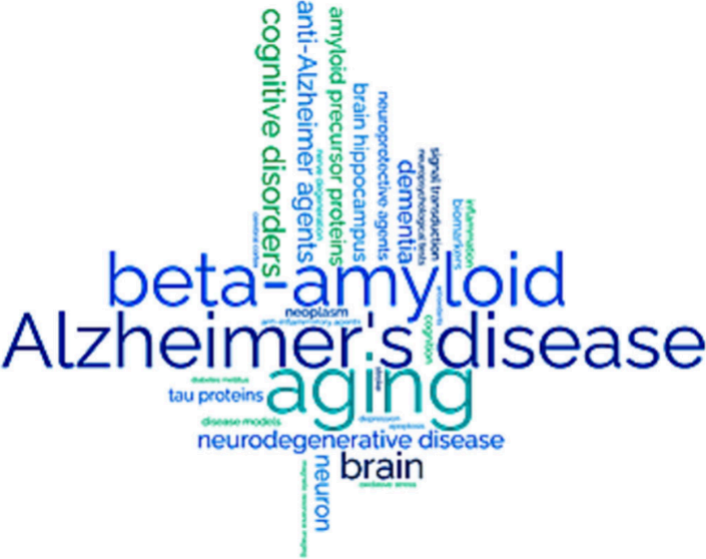

Alzheimer’s disease (AD) is a progressive neurodegenerative disorder characterized by cognitive decline, memory loss, and impaired daily functioning. The pathology of AD is marked by the accumulation of amyloid beta plaques and tau protein tangles in the brain, along with neuroinflammation and synaptic dysfunction. Genetic factors, such as mutations in APP, PSEN1, and PSEN2 genes, as well as the APOE ε4 allele, contribute to increased risk of acquiring AD. Currently available treatments provide symptomatic relief but do not halt disease progression. Research efforts are focused on developing disease-modifying therapies that target the underlying pathological mechanisms of AD. Advances in identification and validation of reliable biomarkers for AD hold great promise for enhancing early diagnosis, monitoring disease progression, and assessing treatment response in clinical practice in effort to alleviate the burden of this devastating disease. In this paper, we analyze data from the CAS Content Collection to summarize the research progress in Alzheimer’s disease. We examine the publication landscape in effort to provide insights into current knowledge advances and developments. We also review the most discussed and emerging concepts and assess the strategies to combat the disease. We explore the genetic risk factors, pharmacological targets, and comorbid diseases. Finally, we inspect clinical applications of products against AD with their development pipelines and efforts for drug repurposing. The objective of this review is to provide a broad overview of the evolving landscape of current knowledge regarding AD, to outline challenges, and to evaluate growth opportunities to further efforts in combating the disease.

## Introduction

Alzheimer’s disease (AD) is a progressive neurological disorder characterized by cognitive decline, memory loss, and changes in behavior and personality, severe enough to strongly interfere with daily life and activities. Alzheimer’s typically begins slowly and worsens over time, eventually leading to severe impairment in memory, reasoning, judgment, and language skills.^1−6^ It is ultimately a fatal form of dementia and substantially shortens life expectancy.^7,8^

Alzheimer’s disease primarily affects neurons, the brain cells responsible for transmitting information throughout the brain and nervous system. The disease is associated with abnormal deposits of proteins in the brain, specifically β-amyloid plaques and tau tangles. These deposits disrupt communication between neurons, a hallmark of the disease.^9^ Advanced age is the greatest risk factor for Alzheimer’s disease, with the majority of cases occurring in individuals over 65. Other risk factors include genetics, family history, and certain lifestyle factors such as cardiovascular health and education level.^10^ Diagnosing AD conclusively involves identifying the hallmark pathological features of the disease, extracellular amyloid-beta plaques and intracellular neurofibrillary tangles composed of hyperphosphorylated tau protein.^11,12^ Diagnosis is typically based on a combination of medical history, cognitive assessments, neurological exams, and ruling out other possible causes of symptoms.^13^ While there is no cure for AD, there are medications and nondrug interventions that can help slow and manage symptoms along with improve quality of life for patients.

AD can be emotionally and physically challenging for both individuals with the disease and their caregivers. Supportive services such as counseling, support groups, and respite care can be valuable resources for managing the impact of the disease on daily life.^14^ Research into AD is intense, with efforts focused on understanding its underlying causes, developing more effective treatments, and ultimately finding a cure. Early detection and intervention are important for maximizing treatment effectiveness and improving outcomes for patients affected by the disease.

In this paper, we overview the research progress in Alzheimer’s disease by analyzing data from the CAS Content Collection.^15^ The CAS Content Collection is the largest human-curated collection of published scientific information, supporting comprehensive quantitative analysis of global research across parameters including time, geography, scientific discipline, application, disease, chemical composition, etc. Covering scientific literature published around the world over the past 150 years in more than 50 languages, the CAS Content Collection encompasses data and discoveries published in more than 50,000 scientific journals and by over 100 patent offices. A major advantage provided by the CAS Content Collection is that, along with the standard reference information, it also provides human curated data on major substances and concepts explored in the scientific publications. The CAS REGISTRY,^16^ the authoritative source for information on more than 250 million unique organic and inorganic substances and 70 million protein and nucleic acid sequences, is part of the CAS Content Collection. The CAS Content Collection is broadly accessible through CAS solutions including CAS SciFinder^17^ and CAS STNext.^18^

Here, we examine the publication landscape in the area in effort to provide insights into current knowledge advances and developments. We review the most discussed and emerging concepts and assess the strategies to combat the disease. We explore the genetic risk factors, pharmacological targets, and comorbid diseases. Finally, we inspect clinical applications of products against AD with their development pipelines and efforts for drug repurposing. The objective of this review is to provide a broad overview of the evolving landscape of current knowledge regarding AD, to outline challenges, and evaluate growth opportunities to further efforts in combating the disease. The merit of the article stems from the extensive, wide-ranging coverage of the most up-to-date scientific information, allowing unique, unmatched breadth of landscape analysis and in-depth insights.

## Overview of Alzheimer’s Disease

### Prevalence and Impact

The prevalence and impact of AD are significant and continue to grow as populations age. According to the World Health Organization (WHO), around 50 million people worldwide have dementia, and AD accounts for 60–70% of cases. The prevalence of Alzheimer’s increases with age, and the majority of cases occur in individuals over 65 years old. As life expectancy increases globally, the number of people living with AD is expected to rise substantially in the coming decades.^19,20^

AD has a profound impact on individuals, families, and society as a whole. Individuals with Alzheimer’s experience a progressive decline in cognitive function, memory loss, and changes in behavior and personality, leading to a loss of independence and ability to perform daily tasks. Caregivers, typically family members, bear a significant burden in providing care and support for individuals with AD, often leading to emotional, physical, and financial strain. The economic impact of AD is substantial, including direct medical costs for diagnosis, treatment, and long-term care, as well as indirect costs associated with lost productivity and caregiver burden.

### Pathogenesis

**Figure 1:**
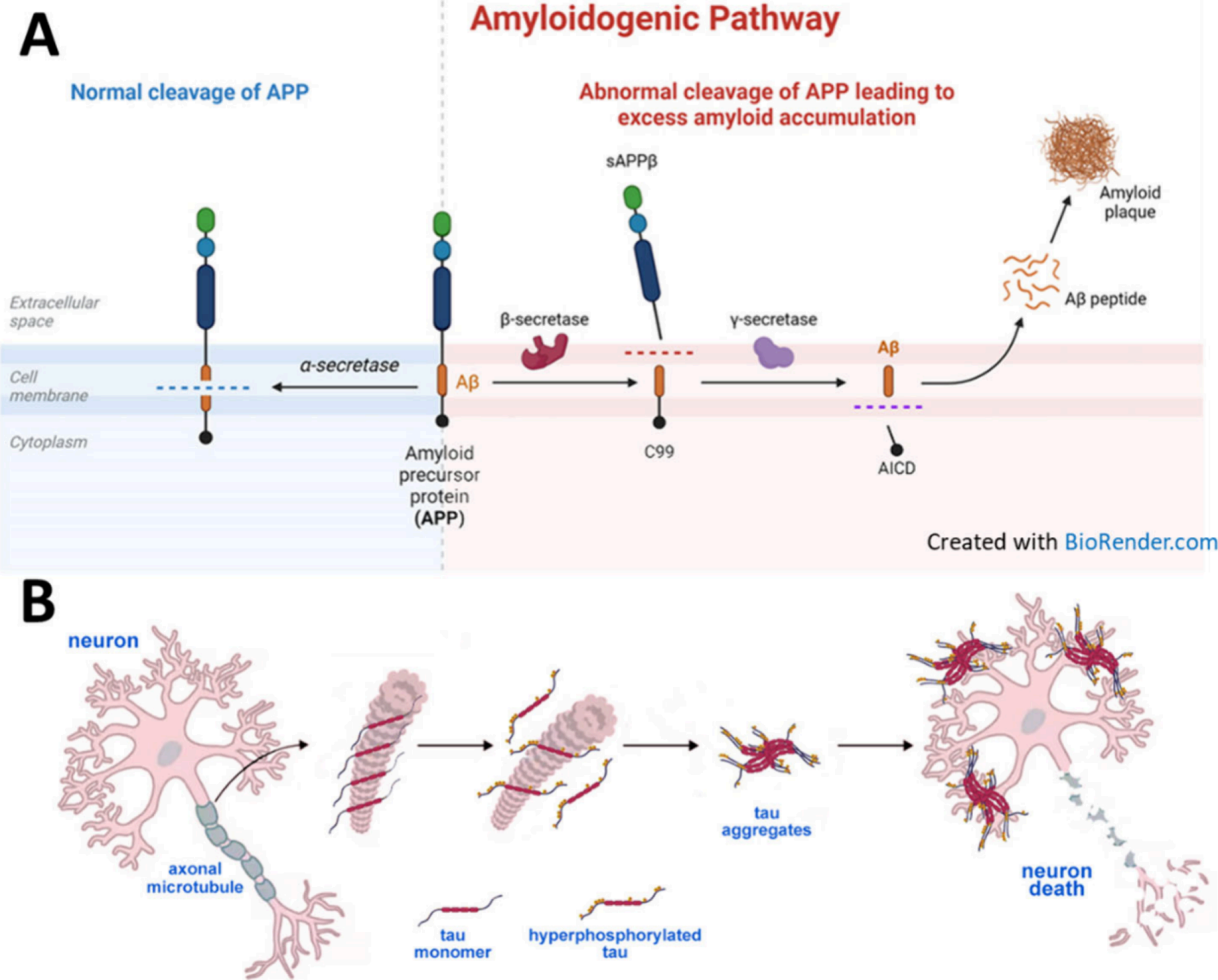
(A) Normal (left) and pathological (right) cleavage of amyloid precursor protein (APP): when cleaved by α-secretase in the middle of the β-amyloid domain (Aβ), it is not amyloidogenic; however, when APP is cleaved by β- and γ-secretase enzymes, neurotoxic Aβ peptides are released, which can accumulate into oligomer aggregates and further form insoluble β-sheet amyloid fibrils triggering local inflammatory response (Created with BioRender.com). C99: 99-residue transmembrane fragment of APP, which is cleaved by β-secretase, is a key step in the amyloidogenic pathway. C99 is then cleaved by γ-secretase to release Aβ and AICD. AICD: an AβPP intracellular domain fragment that is generated by γ-secretase cleavage. Data suggests that AICD mainly comes from C99. AICD, along with C99 and Aβ peptides, may contribute to Alzheimer’s disease pathology. The amyloidogenic pathway mainly takes place in endosomes. (B) Formation of neurofibrillary tangles by the tau protein and subsequent neuron death in tauopathies, such as Alzheimer’s disease. In pathologies, tau becomes hyperphosphorylated and detaches from microtubules, which causes microtubule destabilization; phosphorylated tau aggregates to form neurofibrillary tangles.

The pathogenesis of AD involves a complex interplay of genetic, environmental, and age-related factors, leading to progressive neurodegeneration and cognitive decline.^21^ One of the defining features of AD is the accumulation of β-amyloid protein fragments in the brain, leading to the formation of insoluble plaques.^22,23^ β-Amyloid is produced from the cleavage of a larger protein called amyloid precursor protein (APP) by enzymes known as β-secretase and γ-secretase.^24^ The C99 domain of the amyloid precursor protein (APP) and the amyloid-β protein precursor intracellular domain (AICD) are both involved in the amyloidogenic pathway, which is associated with Alzheimer’s disease. Abnormal processing of APP, along with impaired clearance of β-amyloid from the brain, results in the accumulation of β-amyloid plaques, which are toxic to neurons and disrupt synaptic function (Figure 1A).

Another characteristic feature of AD is the abnormal accumulation of tau protein in the form of neurofibrillary tangles within neurons.^25−27^ Tau protein plays a crucial role in stabilizing microtubules, which are essential for maintaining the structure and function of neurons. In AD, tau protein becomes hyperphosphorylated, leading to its misfolding and aggregation into neurofibrillary tangles (Figure 1B). These tangles interfere with intracellular transport and contribute to neuronal dysfunction and cell death.

The accumulation of β-amyloid plaques and tau protein tangles disrupts normal neuronal function, leading to synaptic dysfunction and impaired neurotransmission.^28^ As the disease progresses, neurons become increasingly vulnerable to damage and eventually undergo cell death, resulting in widespread neuronal loss, particularly in brain regions critical for memory and cognitive function, such as the hippocampus and cerebral cortex.^29^

The structural elucidation of amyloid beta (Aβ) plaques and misfolded tau proteins has significantly advanced our understanding of AD pathology.^11,30−33^ Emerging evidence suggests that AD-related brain changes may result from a complex interplay among abnormal tau and beta-amyloid proteins. Recent advancements in cryo-EM and solid-state NMR have allowed high-resolution imaging of Aβ fibrils.^32,34−36^ This has revealed detailed arrangements of β-sheets and how they stack to form fibrils. Different strains of Aβ fibrils, known as polymorphs, have been identified. These polymorphs may influence the disease’s progression and severity.^37,38^ Dual Inhibitors of amyloid-β and tau aggregation with amyloid-β disaggregating properties were recently suggested as new multifunctional ligands that would affect different stages of AD pathogenesis.^31^ Tauvid has been approved by the US FDA for PET imaging tau pathology in AD.^39^

Neuroinflammation, characterized by the activation of microglia and astrocytes, plays a significant role in the pathogenesis of AD. Chronic inflammation in the brain exacerbates neuronal damage and contributes to disease progression by releasing pro-inflammatory cytokines, reactive oxygen species, and other neurotoxic molecules.^40,41^

Vascular dysfunction and impaired cerebral blood flow are common features of AD and may contribute to neuronal damage and cognitive decline. Chronic cerebral hypoperfusion, resulting from vascular pathology, can exacerbate β-amyloid deposition and tau pathology and increase the risk of cognitive impairment.^42,43^

While most cases of AD are sporadic, a small percentage are inherited in an autosomal dominant fashion, known as familial Alzheimer’s disease (FAD).^44^ FAD typically manifests as early-onset Alzheimer’s disease (EOAD), meaning symptoms often appear before the age of 65, sometimes as early as in a person’s 30s or 40s. Mutations in genes such as amyloid precursor protein (APP), presenilin 1 (PSEN1), and presenilin 2 (PSEN2) are associated with early-onset FAD, leading to increased production or altered processing of β-amyloid.^45^ The apolipoprotein E (APOE) ε4 allele is the strongest genetic risk factor for late-onset Alzheimer’s disease (LOAD) and is associated with increased β-amyloid deposition and enhanced risk of developing AD.

Stress, depression, and anxiety are increasingly recognized as significant risk factors for AD.^46−48^ Chronic stress leads to prolonged elevated levels of cortisol, a stress hormone.^49^ High cortisol levels can damage the hippocampus, a critical brain area for memory and learning, which is often one of the first regions affected in AD. Stress also triggers an inflammatory response, which can lead to chronic neuroinflammation.^50^ This inflammation can contribute to neuronal damage and the progression of AD.^51,52^ Stress has been associated with an increase in amyloid-beta production and tau protein hyperphosphorylation, both of which are hallmark features of AD pathology.^53,54^

Depression is linked to a reduction in hippocampal volume.^55,56^ Since the hippocampus is crucial for memory formation, its atrophy can predispose individuals to cognitive decline and AD. Depression often involves imbalances in neurotransmitters such as serotonin, norepinephrine, and dopamine.^57^ These imbalances can affect cognitive function and increase the risk of AD. Depression is also associated with increased levels of pro-inflammatory cytokines.^58^ Chronic inflammation can exacerbate neurodegeneration and amyloid plaque formation.^59,60^

Similar to stress, anxiety leads to dysregulated cortisol production, which can have detrimental effects on brain structures involved in memory and cognition.^61^ Anxiety can result in changes to synaptic plasticity and neurotransmitter release, impairing cognitive processes and potentially accelerating the onset of AD.^62,63^ Anxiety also increases oxidative stress, leading to cellular damage in the brain. Oxidative damage is a known contributor to the pathogenesis of AD.^64,65^

These conditions often co-occur and can exacerbate each other, creating a vicious cycle that heightens the risk for Alzheimer’s disease. For instance, chronic stress can lead to depression, and depression can increase anxiety, all of which collectively increase AD risk^46^ through shared and overlapping pathways: (i) the hypothalamic-pituitary-adrenal (HPA) axis, which regulates stress responses, can become dysregulated in all three conditions, leading to sustained high levels of stress hormones;^66^ (ii) stress, depression, and anxiety negatively impact neuroplasticity, the brain’s ability to adapt and reorganize, which is vital for maintaining cognitive function;^67,68^ (iii) all three conditions can lead to chronic neuroinflammation, which plays a critical role in the neurodegenerative processes underlying AD.^69,70^

Risk factors for cardiovascular disease, such as hypertension, high cholesterol, diabetes, and obesity, are also associated with an increased risk of AD.^71−74^ Regular physical exercise has been shown to have a protective effect against AD and cognitive decline.^67,75^ Some dietary patterns, such as the Mediterranean diet, rich in fruits, vegetables, whole grains, fish, and healthy fats, may lower the risk of AD.^76−78^ Higher levels of education and engagement in mentally stimulating activities throughout life may help reduce the risk of developing AD.^79,80^

Some environmental factors, such as air pollution, heavy metal exposure, and certain toxins, have been implicated as potential risk factors for AD, although more research is needed to fully understand their impact.^81,82^

### Genetic Background

The genetic background of Alzheimer’s disease (AD) encompasses both rare familial forms and more common late-onset forms.^83−85^

Familial Alzheimer’s disease (FAD) represents a small percentage (∼5–10%^85−87^) of all AD cases and is inherited in an autosomal dominant pattern, meaning that an affected individual has a 50% chance of passing the mutated gene to their offspring.^88,89^ Mutations in three genes have been identified as causative for FAD: (i) Amyloid precursor protein (APP): mutations in the APP gene, located on chromosome 21, tend to inhibit cleavage by α-secretase and facilitate preferential cleavage by β-secretase, which leads to increased production or altered processing of β-amyloid, resulting in the accumulation of amyloid plaques in the brain; (ii) Presenilin 1 (PSEN1): mutations in the PSEN1 gene, located on chromosome 14, are the most common cause of FAD. PSEN1 is a component of the γ-secretase complex involved in the cleavage of amyloid precursor protein (APP), and mutations in PSEN1 enhance cleavage by γ-secretase and lead to increased production of β-amyloid; (iii) Presenilin 2 (PSEN2): mutations in the PSEN2 gene, located on chromosome 1, are less common but can also cause FAD. Like PSEN1, PSEN2 is involved in the processing of APP, enhanced cleavage by γ-secretase, and the production of β-amyloid.^24,90−92^

Late-onset Alzheimer’s disease (LOAD) is the most common form of AD and typically occurs after age of 65.^93,94^ While LOAD has a strong genetic component, it is influenced by multiple genetic and environmental factors. The strongest genetic risk factor for LOAD is the apolipoprotein E (APOE) gene, located on chromosome 19. The APOE gene has three common alleles: ε2, ε3, and ε4. The ε4 allele of APOE (APOE4) is associated with an increased risk of developing AD and a younger age of onset, at 65.1, with a narrow 95% prediction interval.^95^ Individuals who inherit one copy of the APOE ε4 allele have an increased risk, while those who inherit two copies (homozygous) have an even higher risk, estimated to be 8 to 12 times greater than those with no ε4 alleles.^84,96^ Recent study has concluded that APOE4 homozygotes represent in fact a distinct genetic form of AD.^95,97^

In addition to APOE, several other genetic variants have been identified as risk factors for LOAD through genome-wide association studies. These include genes involved in inflammation, cholesterol metabolism, immune response, and synaptic function. While these genetic risk factors increase susceptibility to AD, they do not guarantee that an individual will develop the condition. Environmental factors and gene-environment interactions also play a significant role in disease risk.^93,94,98,99^

### Symptoms and Progression

AD progresses gradually over time, and the symptoms can vary from person to person. One of the most common early signs is difficulty remembering recent events, conversations, or information. Individuals may have trouble with tasks that require planning, decision-making, and problem-solving. They may become disoriented, especially in unfamiliar environments, and have difficulty following directions or understanding the passage of time. They may have trouble finding the right words or understanding spoken or written language. Changes in mood, such as depression, anxiety, or apathy, and shifts in personality traits may occur.^100−104^

As AD progresses, memory loss becomes more severe and may include forgetting the names of close family members or important personal information. Language difficulties may worsen, making it increasingly difficult to engage in conversations or express thoughts. Individuals may have difficulty making decisions or solving problems, and they may exhibit poor judgment in everyday situations. Behavioral symptoms such as agitation, aggression, wandering, or social withdrawal may become more pronounced. In the later stages of AD, individuals may experience physical symptoms such as difficulty swallowing, walking, or performing basic self-care tasks. As the disease progresses, individuals become increasingly dependent on others for assistance with activities of daily living.^8,104,105^

In the advanced stages of AD, individuals may lose the ability to communicate verbally, recognize loved ones, or control movement. They may require round-the-clock care in a residential facility or at home with the assistance of caregivers. Physical complications such as infections, falls, and malnutrition become more common, contributing to further decline in health.^104−106^

### Diagnosis

Diagnosing AD involves a comprehensive assessment by healthcare professionals, including medical history, physical examination, cognitive assessments, and imaging tests. Cognitive tests are used to evaluate memory, attention, language, problem-solving, and other cognitive functions. These assessments may include standardized tests such as the Mini-Mental State Examination (MMSE) or the Montreal Cognitive Assessment (MoCA).^107−109^ The healthcare provider may also administer more in-depth neuropsychological testing to assess specific cognitive domains and identify patterns of impairment.

A neurological examination may be performed to assess for signs of neurological dysfunction, such as abnormal reflexes, muscle weakness, or coordination problems. Imaging tests such as magnetic resonance imaging (MRI) or positron emission tomography (PET) scans may be used to rule out other possible causes of cognitive impairment, such as stroke, tumor, or hydrocephalus.^110^ These imaging studies can also help visualize changes in the brain associated with AD, such as atrophy (shrinkage) of brain regions involved in memory and cognition or the presence of β-amyloid plaques and tau tangles. Blood tests may be performed to rule out other medical conditions that can cause cognitive impairment, such as thyroid dysfunction, vitamin deficiencies, or infections.^110^ Cerebrospinal fluid (CSF) analysis may be considered in some cases to measure levels of β-amyloid and tau proteins, which can be indicative of AD pathology. In addition, noninvasive diagnostic tests are being considered as a part to the comprehensive AD assessment. One such test, PrecivityAD2 is a clinical care assay that measures both plasma amyloid beta and tau peptide concentrations.^111,112^ Other assays utilizing not only blood, but saliva and urine are currently being researched.^113,114^

Diagnosis of AD is typically based on clinical criteria established by organizations such as the National Institute on Aging and the Alzheimer’s Association (NIA-AA) or the International Working Group (IWG) for AD.^115−117^ These criteria consider the presence and pattern of cognitive symptoms, the progression of symptoms over time, and the exclusion of other possible causes of dementia.

### Treatment and Management

Treatment and management of AD aim to alleviate symptoms, slow down the progression of the disease, and improve the quality of life for individuals affected by the condition. While there is currently no cure for AD, various interventions can help manage symptoms and support overall well-being.^118−121^

Medications such as donepezil (Aricept), rivastigmine (Exelon), and galantamine (Razadyne) are cholinesterase inhibitors commonly prescribed to treat cognitive symptoms associated with AD.^122^ These drugs work by increasing levels of acetylcholine, a neurotransmitter involved in memory and learning, in the brain.^123,124^ Memantine (Namenda) is another medication approved for the treatment of AD.^125^ It works by regulating the activity of glutamate receptors, another neurotransmitter involved in learning and memory.^126,127^ These medications may help delay the decline in cognitive function, behavior, and daily functioning in some individuals with AD. Selective serotonin reuptake inhibitors (SSRIs) are primarily known for their use in treating depression and anxiety disorders. However, they have also been investigated for their potential benefits in managing symptoms of AD.^128,129^ SSRIs work by increasing the levels of serotonin in the brain by inhibiting its reuptake into the presynaptic cell, making more serotonin available to bind to the postsynaptic receptor.^130,131^ Commonly used SSRIs in AD include citalopram, sertraline, fluoxetine and escitalopram.^130−133^ These SSRIs are also used to manage depression and anxiety in AD, although their specific effects on AD-related symptoms are less well-documented.^134^

US FDA has approved a few antiamyloid-beta monoclonal antibodies for the treatment of Alzheimer’s disease.^135−137^ These therapies aim to reduce amyloid-beta plaques in the brain, which are thought to play a crucial role in the progression of AD. These monoclonal antibody drugs include: (i) Aducanumab (Aduhelm),^138^ targeting aggregated forms of amyloid-beta, including soluble oligomers and insoluble fibrils–by binding to these aggregates, it promotes their clearance from the brain (planned to be discontinued in November 2024, for “reprioritization” of its resources to focus on other Alzheimer’s disease treatments^139^); (ii) Lecanemab (Leqembi)^140^ that preferentially binds to soluble amyloid-beta protofibrils, facilitating their clearance from the brain–this action helps reduce amyloid plaque buildup and is thought to slow the progression of AD; (iii) Donanemab,^141^ which targets a specific modified form of amyloid-beta called N 3pG, which is found in amyloid plaques–by binding to this form of amyloid-beta, donanemab promotes the clearance of amyloid plaques from the brain. The approval of these antiamyloid-beta monoclonal antibodies marks a significant milestone in AD treatment.^142^ These therapies offer hope for slowing disease progression by targeting one of its key pathological features. However, the clinical benefits, optimal use, and safety profiles of these treatments continue to be areas of active research and discussion in the medical community. For more information on AD medications, see Table 1 and Figure 10 in the Landscape analysis section further in the text.

**Table 1:**
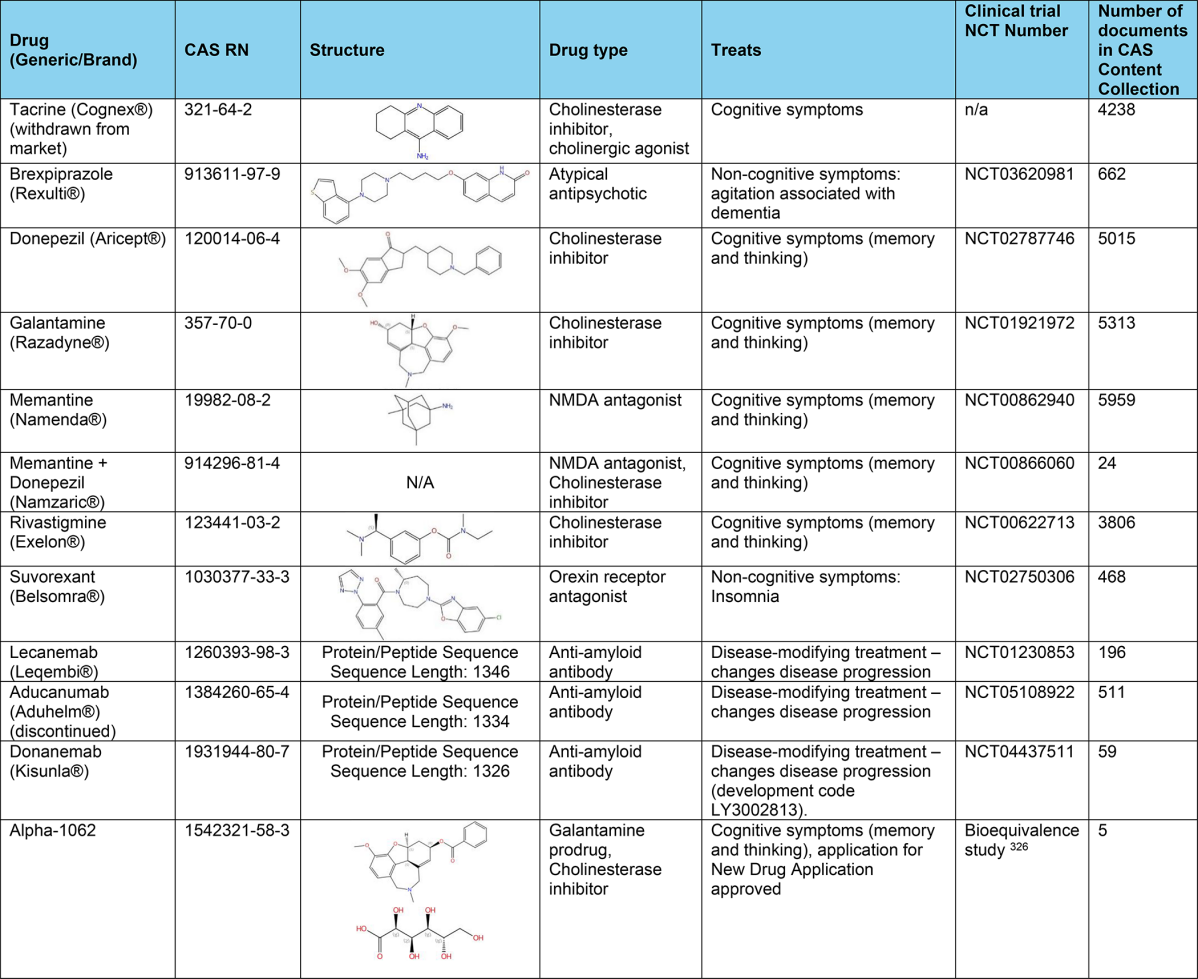
FDA Approved Medications That Mitigate Symptoms of Alzheimer’s Dementia or Change the Disease Progression^120,124,323−326^

Engaging in mentally stimulating activities may help maintain cognitive function and slow down cognitive decline. Regular physical activity has been shown to have beneficial effects on cognition and overall health in individuals with AD. Eating a balanced diet can support overall brain health and well-being. Addressing sleep disturbances and establishing healthy sleep habits can help improve cognitive function and mood in individuals with AD.^143−145^

Behavioral interventions such as cognitive-behavioral therapy (CBT) or behavior modification techniques may help manage challenging behaviors such as agitation, aggression, or wandering.^146−148^ Counseling, support groups, and other psychosocial interventions can provide emotional support, education, and coping strategies for individuals with AD and their caregivers.^149,150^

## Landscape Analysis of Alzheimer’s Disease Research

### Journal Publication and Patent Trends (CAS Content Collection Data)

**Figure 2:**
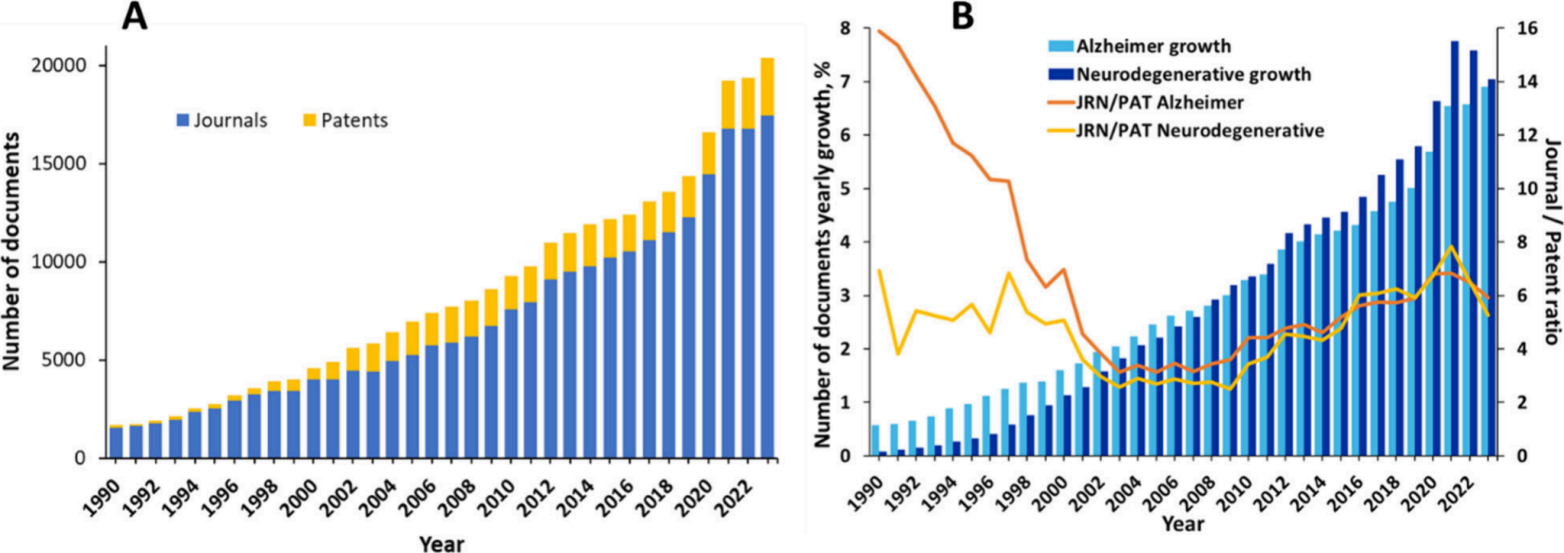
(A) Yearly trend of the number of documents (journal articles and patents) in the CAS Content Collection related to the AD. (B) Comparison between relative growth in the number of documents related to the AD (dark blue bars) and all neurodegenerative diseases (light blue bars); orange and yellow lines compare the journal (JRN)/patent (PAT) ratio for the AD and all neurodegenerative diseases, respectively.

Our search in the CAS Content Collection^15^ for AD-related documents (search term: Alzheimer* in Title, Abstract, Keywords, and Concept fields) retrieved over 300,000 scientific documents (mainly journal articles and patents), including over 250,000 articles in scientific journals and nearly 50,000 patents. There has been a steady growth of these documents over the last three decades, with an >30% increase in the last three years (2021–2023) (Figure 2A). The journal articles largely dominate, showcasing the intense research in the area. The relative growth in the number of documents related to the AD as well as the journal/patent number ratio virtually coincides with that for the overall class of neurodegenerative diseases during the last two decades (Figure 2B) which is understandable since AD is by far the most prevalent and most widely explored disease in that class of diseases. The high journal/patent number ratio with regards to the AD research in the 1990s reflect the initial period of knowledge accumulation preceding the subsequent opportunities for commercialization.

**Figure 3:**
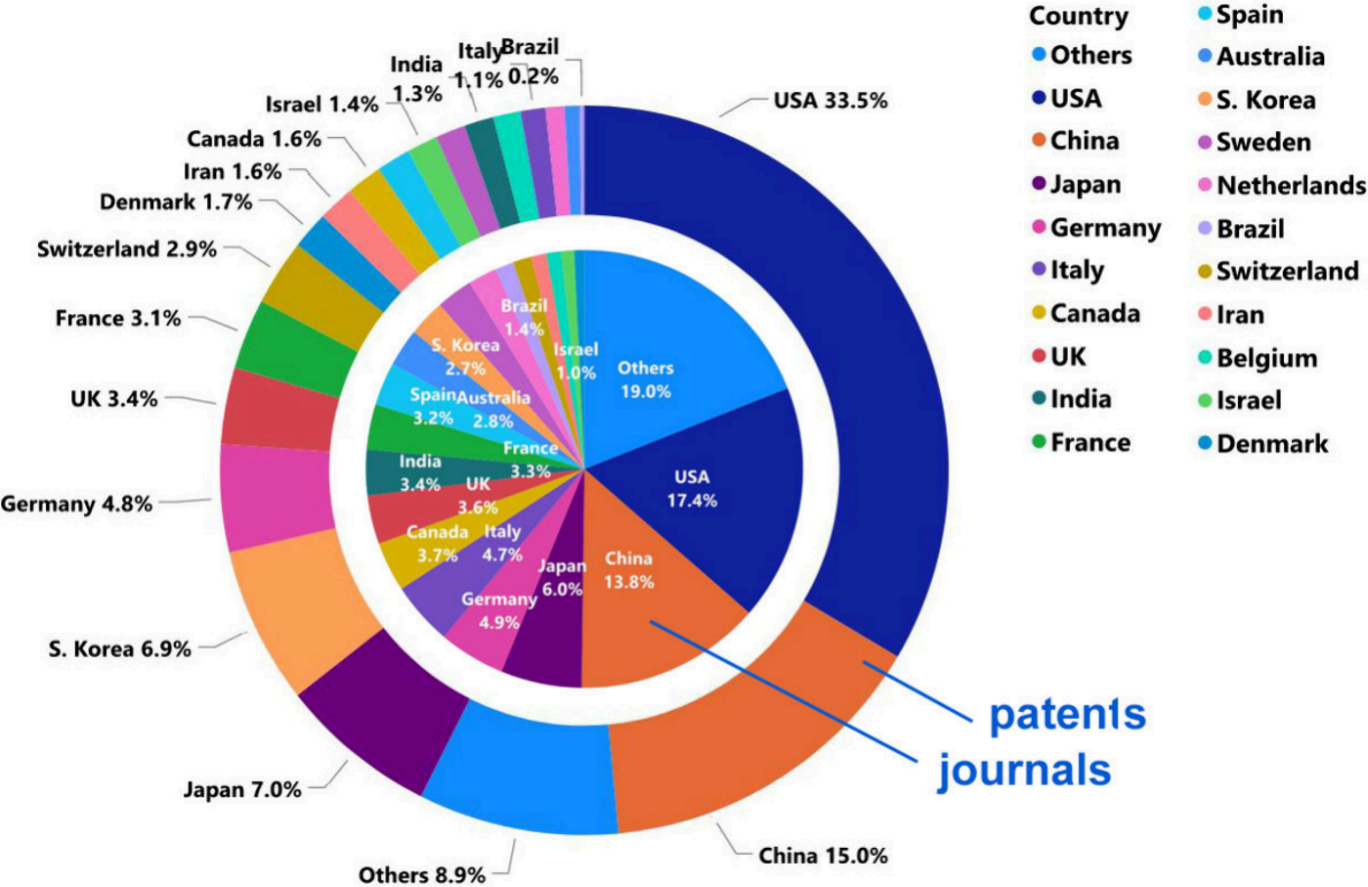
Top countries/regions with respect to the number of AD-related journal articles (inner pie chart) and patents (outer donut chart) in the CAS Content Collection.

**Figure 4:**
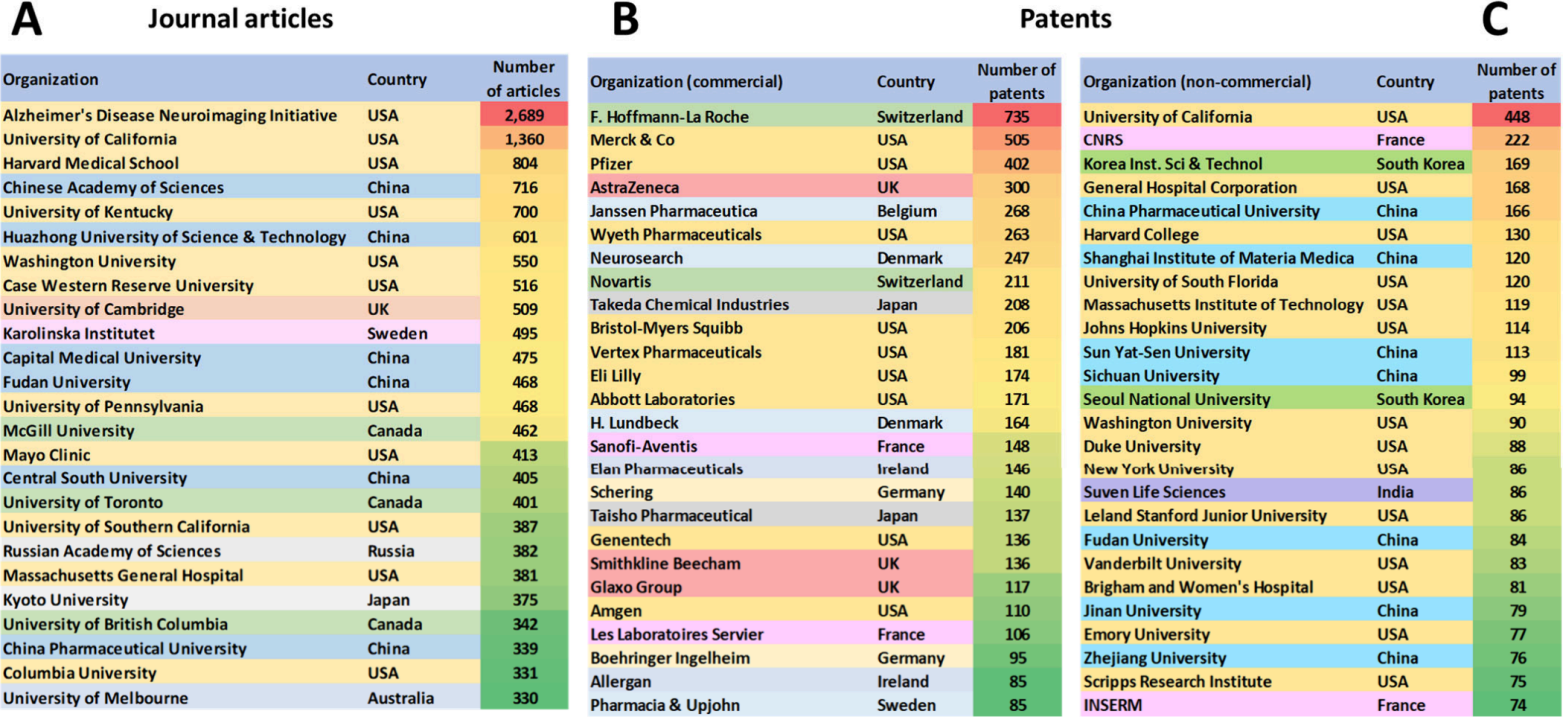
Leading organizations publishing documents related to AD as found in the CAS Content Collection: (A) journal articles, (B) patents by commercial organizations, (C) patents by noncommercial organizations.

**Figure 5:**
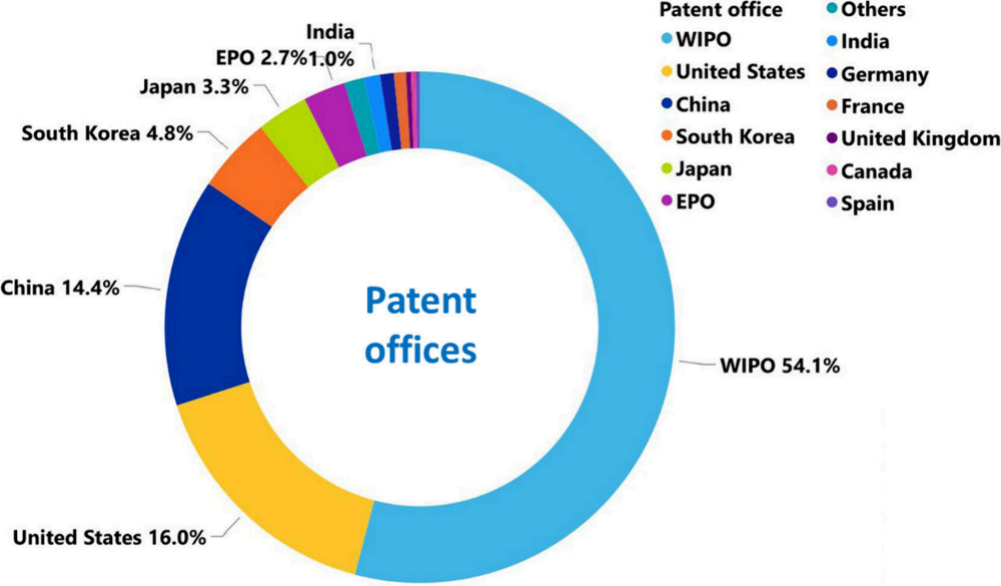
Distribution of patents related to AD with respect to patent offices they have been filed at (WIPO, World Intellectual Property Organization; EPO, European Patent Office).

**Figure 6:**
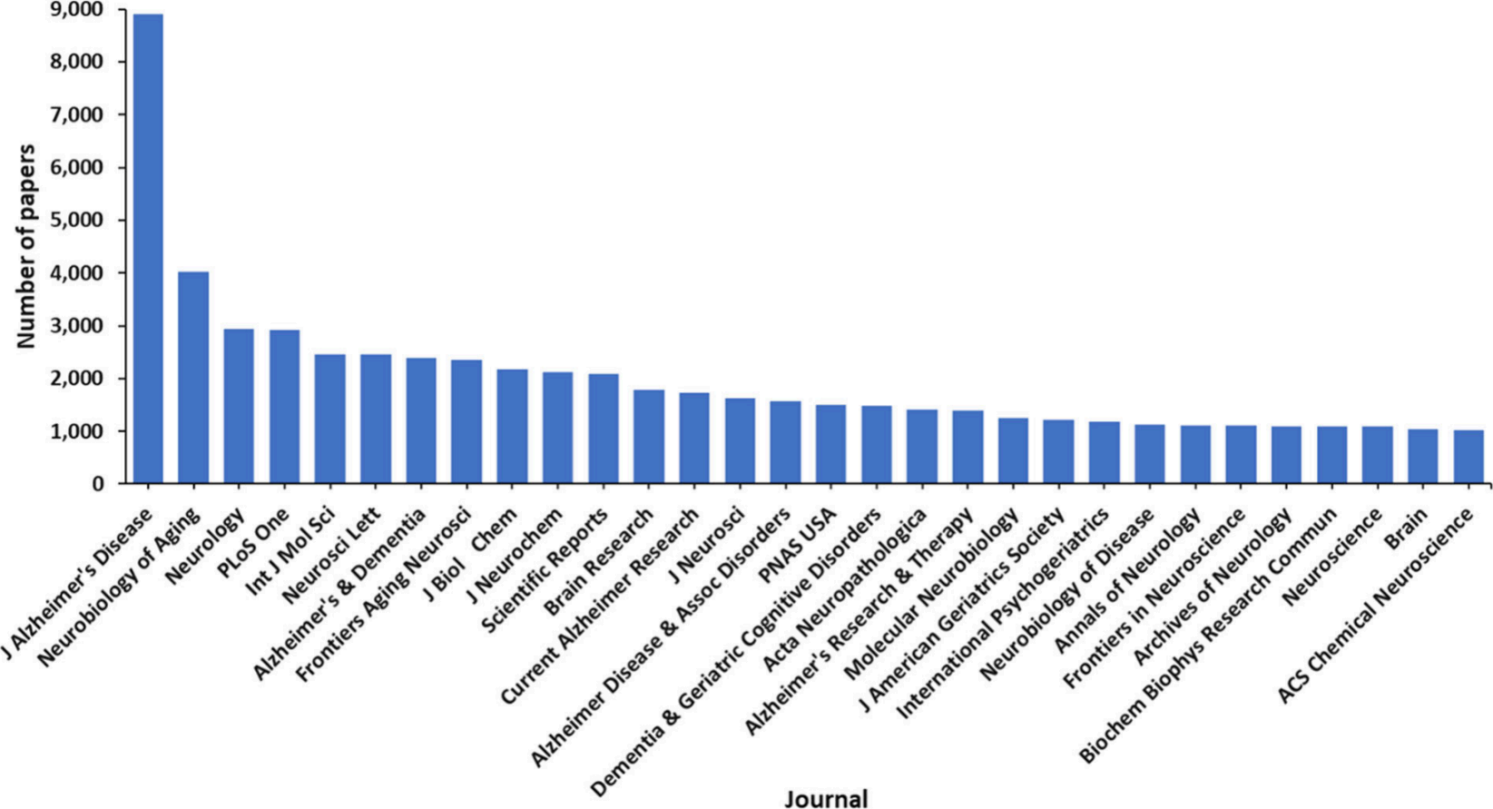
Leading scientific journals publishing articles related to AD as found in the CAS Content Collection.

**Figure 7:**
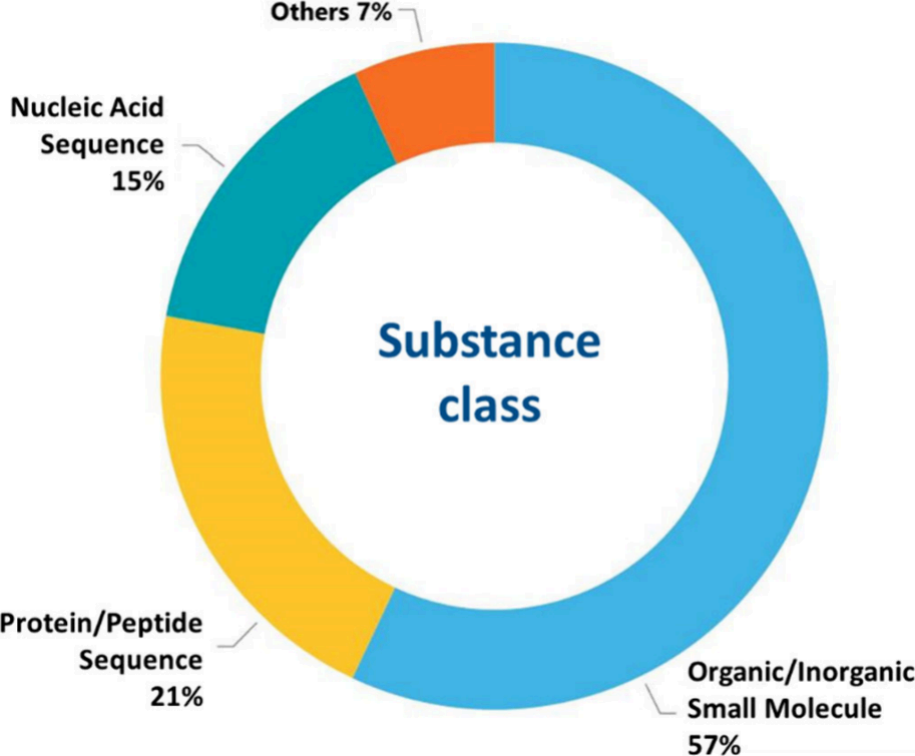
Distribution of the major substance classes between the documents related to the AD research.

USA, China, Japan, Germany, and South Korea are the leaders with respect to the number of published journal articles and patents related to AD research (Figure 3). The Alzheimer’s Disease Neuroimaging Initiative, University of California, Harvard Medical School, and the Chinese Academy of Sciences are the leaders with respect to the number of published journal articles related to the AD (Figure 4A). Patenting activity is dominated by corporate players as compared to academics (Figure 4B,C). F. Hoffmann-La Roche, Merck, Pfizer, and AstraZeneca have the highest number of patent applications among the companies (Figure 4B), while the University of California, the Centre national de la recherche scientifique (CNRS, France), and the Korea Institute of Science and Technology lead among the noncommercial organizations (Figure 4C). The most patent applications have been filed at the World Intellectual Property Organization (WIPO) followed by the US and China patent offices (Figure 5). *Journal of Alzheimer’s Disease* is a distinct leader with respect to the number of published articles related to Alzheimer’s disease research, followed by *Neurobiology of Aging*, *Neurology*, and *PLoS One* (Figure 6). With respect to the substance classes explored in the AD-related documents in CAS Content Collection, the largest part belong to the organic and inorganic small molecules (Figure 7).

### Aging and β-Amyloid Are the Most Widely Explored Concepts in AD Research

**Figure 8:**
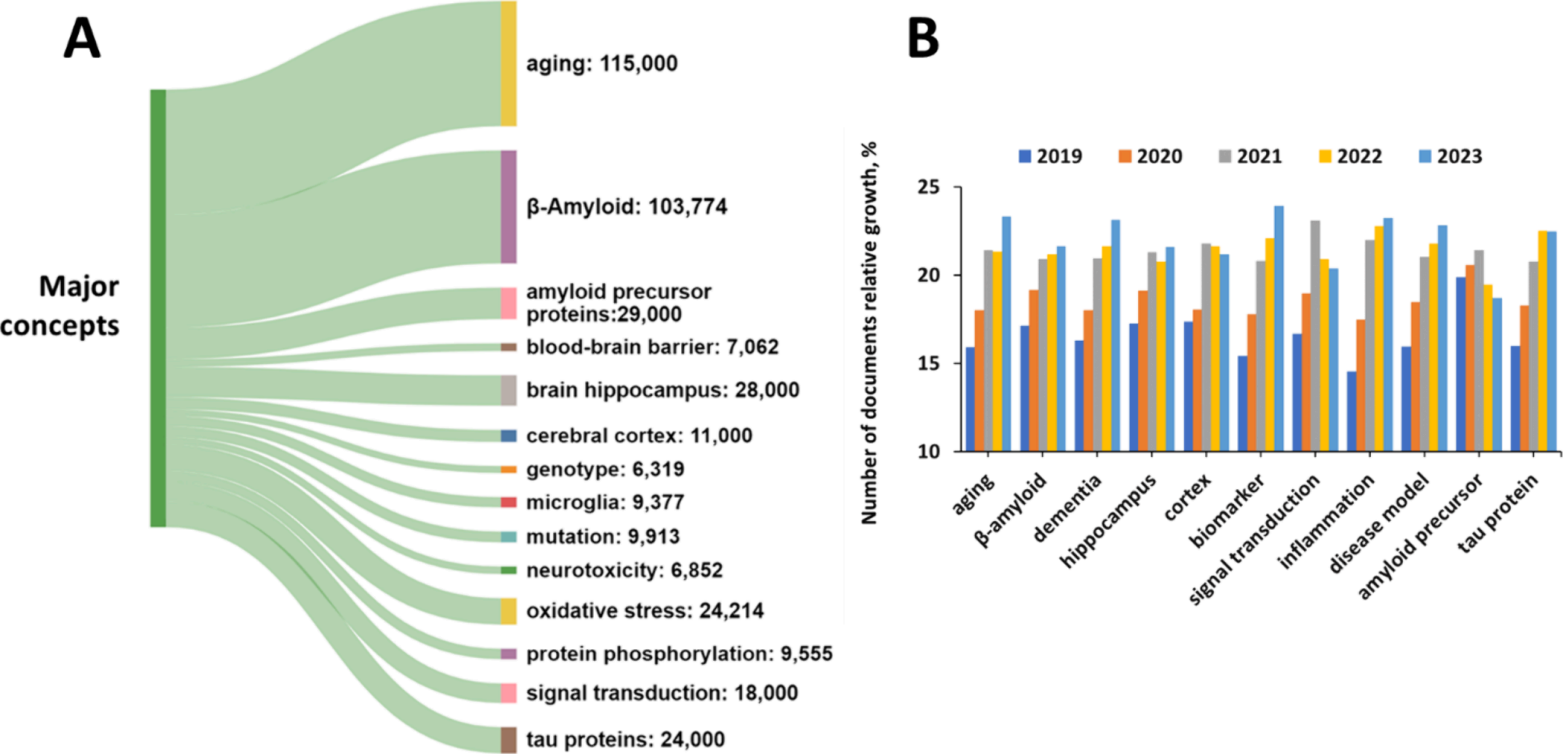
(A) Key concepts explored in the scientific publications related to AD as found in the CAS Content Collection, with a number of documents indicated. (B) Yearly growth of the number of documents (journal articles and patents) exploring certain essential AD-related concepts.

Utilizing the key advantage of the CAS Content Collection in providing information on major substances and concepts explored in the scientific publications as identified by subject matter experts (SME), we were able to identify most frequently discussed concepts within the Alzheimer’s disease-related document subset of the database, as well as to perform cross-concept searches in order to identify concept co-occurrence in the documents. The distribution of the Alzheimer’s disease-related concepts in the published documents (journals and patents) and their annual trends are presented in Figure 8. Aging and β-amyloid are the most widely explored concepts (Figure 8A). This finding is well rationalized. Indeed, aging is a leading risk factor for Alzheimer disease.^20,151,152^ Biological processes altered with aging, which have been implicated in AD. It has been explicated that the pathogenesis of AD and other neurodegenerative diseases is associated with the major hallmarks of aging: genomic instability, telomere attrition, epigenetic alterations, loss of proteostasis, mitochondrial dysfunction, cellular senescence, deregulated nutrient sensing, stem cell exhaustion and altered intercellular communication.^153−156^

According to the existing hypotheses, the accumulation of toxic amyloid β-protein (Aβ) in the central nervous system is the major pathophysiological feature and the basis of AD.^157−159^ Extracellular abnormalities of Aβ levels in the brain may result in the accumulation of Aβ, forming a structure rich in β-sheet structures. Upon forming oligomers, they recombine into fibrils to form amyloid plaques (Figure 1).

### Biomarkers Are the Fastest Growing Concept Related to AD

Figure 8B illustrates the annual trends in some key concepts during the last five years (2019–2023). The biomarker concept appears as the fastest growing one. Biomarkers are measurable indicators or characteristics that can provide information about the presence, progression, or risk of developing the disease.^160,161^ They play a crucial role in diagnosing, monitoring disease progression, assessing treatment response, and facilitating research into the underlying mechanisms of disease. For example, the levels of an amyloid-beta peptide, Aβ42, a fragment of amyloid-beta protein, in the cerebrospinal fluid (CSF) are typically decreased in individuals with AD compared to healthy individuals.^162−165^ Aβ42 is known to aggregate and form plaques in the brain, a hallmark pathology of Alzheimer’s. CSF levels of tau proteins, particularly phosphorylated tau, are elevated in AD patients.^164−166^ Tau proteins are involved in microtubule stabilization, and their abnormal phosphorylation leads to neurofibrillary tangle formation, another pathological feature of AD.^25,167^

**Figure 9:**
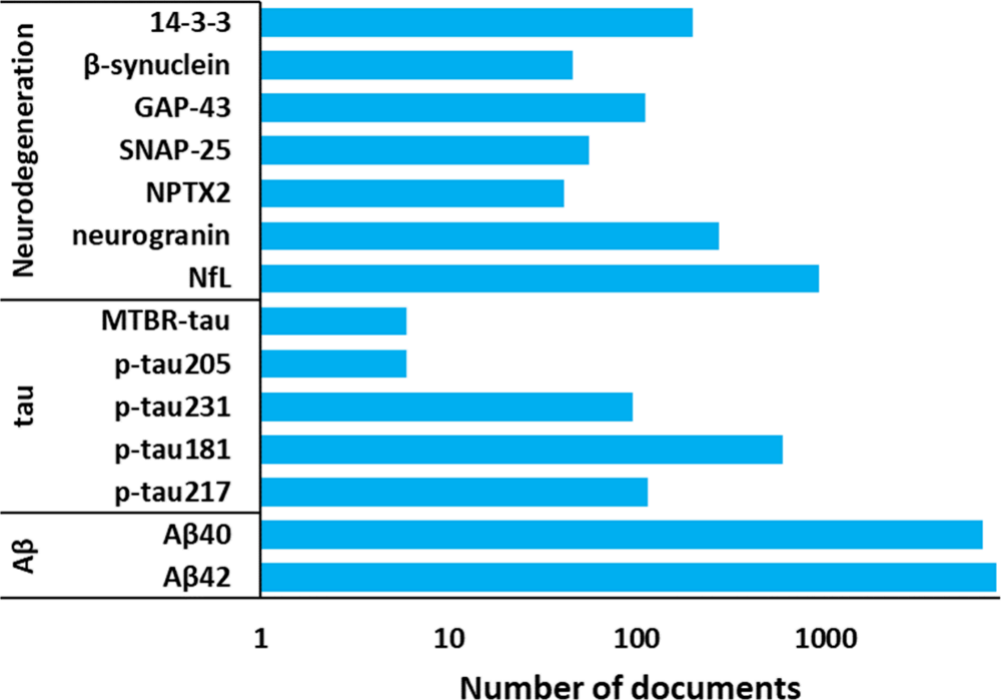
AD biomarkers as represented in the CAS Content Collection.

Overall, biomarkers from cerebrospinal fluid (CSF) and from blood plasma including amyloid peptides and phosphorylated tau are used as AD biomarkers in clinic.^168−171^ The procedure for detection of CSF-based biomarkers is intrusive, it requires a lumbar puncture. In contrast, blood tests for AD biomarkers offer the potential for noninvasive, cost-effective screening and monitoring of disease progression, hence, blood-based biomarkers are highly preferred and are currently being actively researched and developed.^172−174^ These include measures of amyloid-beta, tau, neurofilament light chain, and other proteins associated with AD pathology (Figure 9). Noteworthy, the p-tau217 blood test, for which US FDA granted Breakthrough Device designation in March 2024,^175^ has been reported to exhibit high diagnostic accuracy for identifying AD among individuals with cognitive symptoms.^176^ Genetic variants associated with AD, such as mutations in the amyloid precursor protein, presenilin 1 (PSEN1), and presenilin 2 (PSEN2) genes, can serve as biomarkers for increased risk of developing familial forms of AD.^45,177^ The apolipoprotein E (APOE) gene, particularly the ε4 allele, is a well-established genetic risk factor for late-onset AD. APOE genotyping can help identify individuals at higher risk of developing AD.^178,179^

Positron emission tomography (PET) imaging using radiotracers that bind to amyloid plaques allows for the visualization and quantification of amyloid deposition in the brain.^180−182^ Amyloid PET scans can help confirm the presence of AD pathology in individuals with cognitive impairment. PET imaging using radiotracers specific to tau aggregates enables the visualization of neurofibrillary tangles in the brain, providing insights into the spread and distribution of tau pathology in AD. Magnetic resonance imaging (MRI) techniques can detect structural changes, such as hippocampal atrophy and cortical thinning, as well as functional alterations, such as changes in brain connectivity, associated with AD.^183−185^

Biomarkers of neuroinflammation, such as markers of microglial activation and inflammatory cytokines, are being investigated for their potential role in AD pathogenesis and progression.^186−188^ Biomarkers of neuronal injury and degeneration, such as neurofilament light chain (NFL) levels in CSF or blood, can indicate ongoing neurodegenerative processes in AD.^189,190^ The identification and validation of reliable biomarkers for AD hold great promise for improving early diagnosis, monitoring disease progression, and assessing treatment response in clinical practice. Ongoing research efforts continue to advance our understanding of Alzheimer’s biomarkers and their clinical utility in the management of this devastating neurodegenerative disorder. Exemplary AD biomarkers are illustrated in Figure 9.

### Association of Alzheimer’s Disease with Other Diseases

**Figure 10:**
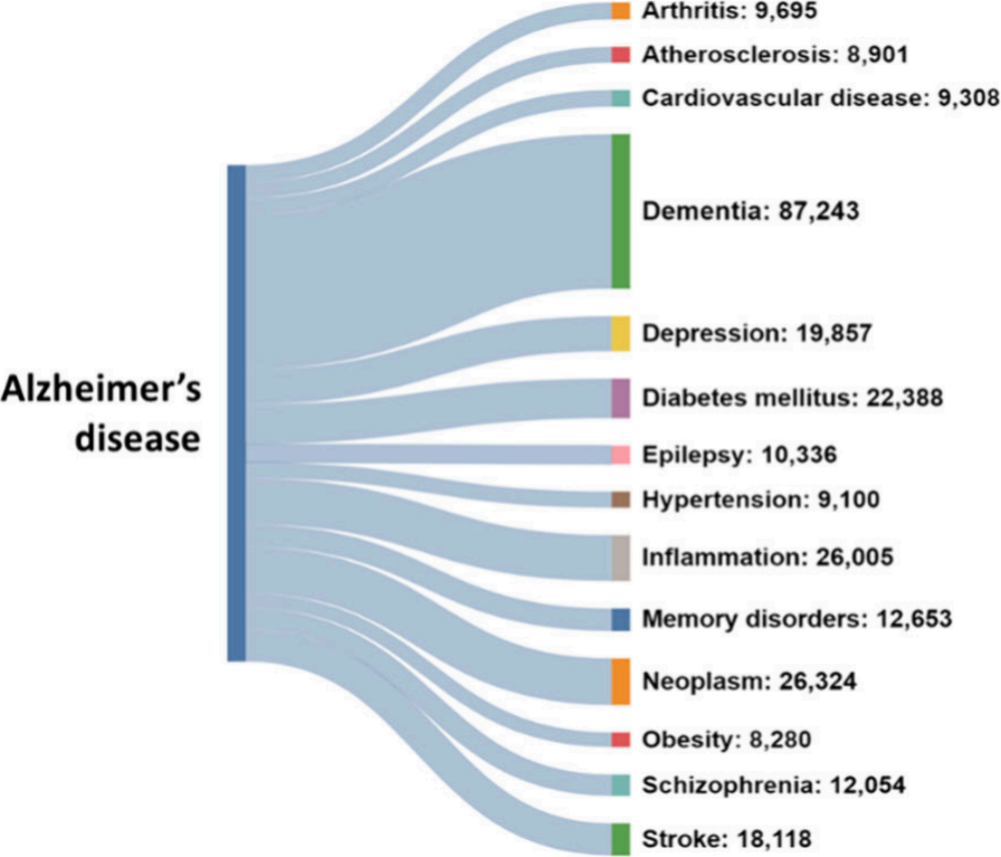
Co-occurrence of the AD concept with other disease concepts in the CAS Content Collection documents, with the associated number of documents shown.

A large assortment of comorbid diseases is associated with AD.^191^ There is evidence that chronic diseases, including diabetes, cardiovascular disease, depression, and inflammatory bowel disease, may be associated with enhanced risk of AD. Disruption in certain shared biological pathways has been suggested as the underlying mechanism for the relationship between AD and these diseases. Particularly, inflammation is a common dysregulated pathway shared by most of the diseases associated with AD. We examined the co-occurrence of certain diseases with AD as reflected by the co-occurrence of the respective concepts in the documents of the CAS Content Collection (Figure 10A). Dementia and inflammation are between the expected co-occurrences. With respect to cancer, inverse occurrence of cancer and Alzheimer disease has been reported: patients with dominant cancer had a 43% lower risk of developing AD, and those with prevalent AD had a 69% lower risk of being diagnosed with cancer.^192−194^ Stroke has been reported to be associated with AD among elderly patients. The relation is strong in the presence of certain vascular risk factors.^195,196^ Recently, there is accumulating evidence demonstrating that hyperglycemia is a potential risk factor for the development of cognitive impairment or AD.^197,198^ It has been reported that AD patients have a high risk of developing certain types of epilepsy and subclinical epileptiform activity.^199^

### Pharmaceutical Targets and Genetic Risk Factors

**Figure 11:**
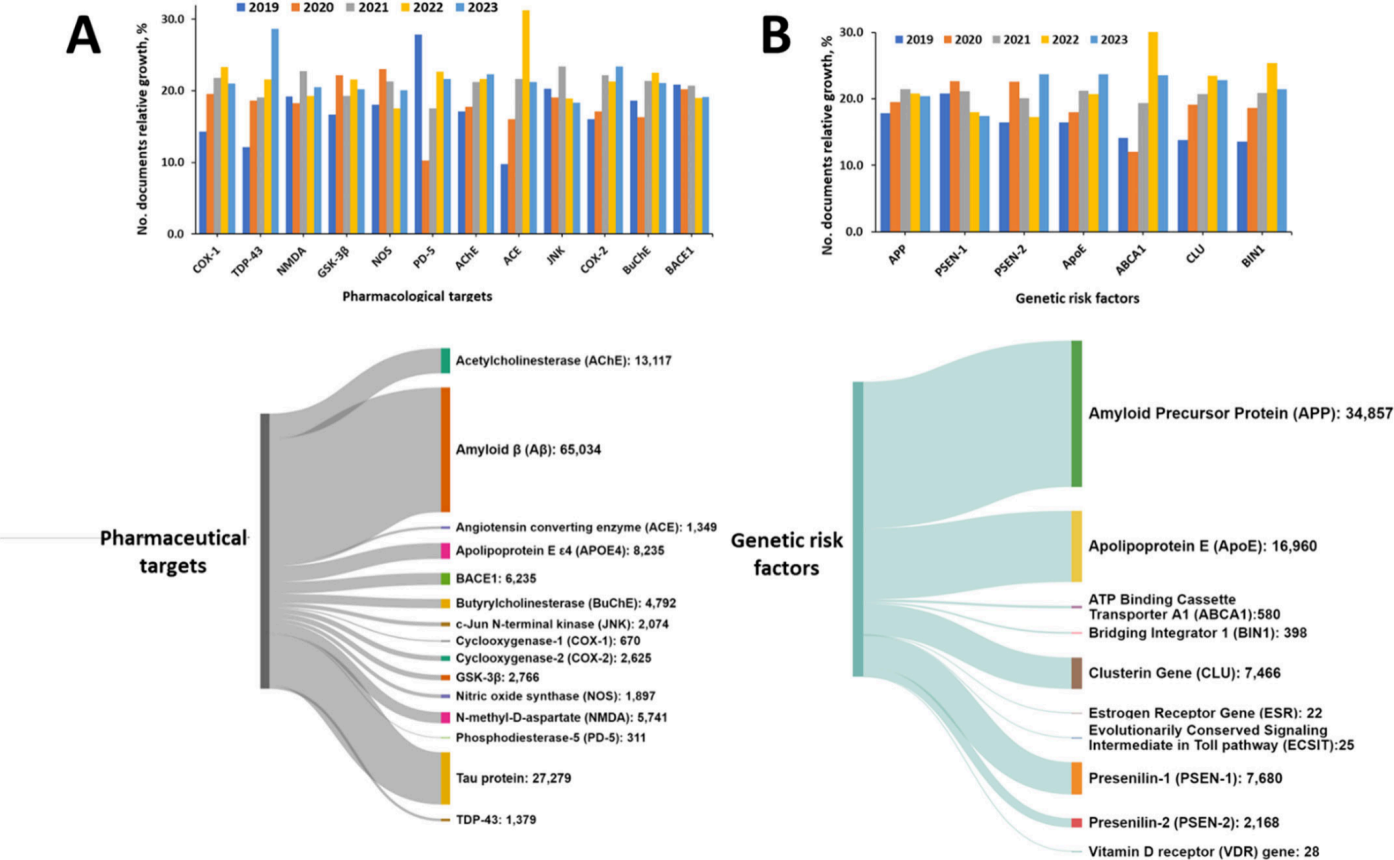
Pharmacological targets (A) and genetic risk factors (B) of AD. Upper panels illustrate the relative growth of the number of documents in the last five years (2019–2023), while the lower panels show the number of documents related to various major target proteins and genetic risk factors.

There are multiple potential pharmaceutical targets for the treatment of AD, as currently documented in the Common Alzheimer’s Disease Research Ontology (CADRO),^200^ including amyloid beta (Aβ) peptide, tau protein, apolipoprotein E4 (APOE4), lipids, lipoprotein and neurotransmitter receptors, enzymes, and many other regulators, and multitarget interventions.^201^ Amyloid beta and tau proteins are the major undisputed pharmacological targets for disease modifying therapies of AD (Figure 11A, lower panel). The fastest growing target according to the CAS data is the transactive response DNA binding protein of 43 kDa (TDP-43), a nuclear protein involved in the regulation of gene expression (Figure 11A, upper panel). Cytoplasmic inclusion bodies comprising phosphorylated and truncated forms of TDP-43 have been found in multiple AD cases, as well as in other proteinopathies including amyotrophic lateral sclerosis, frontotemporal dementia. TDP-43 deposits have been also found in neurons with neurofibrillary tangles. There is emerging evidence that TDP-43 may spread in a prion-like manner, which means it could propagate from cell to cell, potentially contributing to the progression of neurodegenerative diseases like AD.^202^ The most common genetic risk factor for AD, apolipoprotein E4 (APOE4), is associated with increased frequency of TDP-43 pathology.^203−206^

Angiotensin-converting enzyme (ACE) is another pharmacological target of AD exhibiting rapid growth in the number of publications included in the CAS Content Collection (Figure 11A, upper panel). Indeed, among the Aβ degrading enzymes such as neprilysin, insulin-degrading enzyme, endothelin-converting enzyme, and ACE, the last one is the most commonly targeted enzyme by inhibitors, mainly because it plays central role in the regulation of blood pressure and hypertension.^207,208^ However, genetic, pathological and biochemical studies have associated ACE with AD.^209^ ACE has been shown able to convert neurotoxic β-amyloid protein Aβ42 to Aβ40. Aβ42 is believed to play a causative role in the development of AD, whereas Aβ40 exhibits neuroprotective activities against Aβ42 aggregation as well as against metal-induced oxidative damage.^210^

Genetic factors play a major role in the development of AD. In the late-onset type of AD, which applies to 95% of cases, the underlying etiology is supposedly caused by a combination of genetic and environmental factors in the approximate ratio of 70:30, respectively.^211^ In the rare cases of early-onset AD, the disease etiology is allegedly almost exclusively genetic.^212−216^ Hence, genetics seem to play a major role in all types of AD.

Mutations in the dominant genes including Amyloid Precursor Protein (APP), Presenilin-1 (PSEN-1), Presenilin-2 (PSEN-2), apolipoprotein E (ApoE), and others, are associated with AD.^83,217^ From 30 discovered mutations in the APP gene, 25 have been found related to AD and causing accumulation of Aβ.^218,219^ PSEN1 and PSEN2 genes are also the autosomal dominant form of early-onset AD. Mutation in PSEN1 gene is more frequent, with more than 200 mutations, while in the PSEN2 gene only a rare form with less than 40 mutations was identified.^45,220^ Apolipoprotein E (ApoE), especially ApoEε4 plays an important role in Aβ deposition as a senile plaque. It has been found to cause cerebral amyloid angiopathy, known as a marker for AD.^221^ ApoEε4 is also related to vascular damage in the brain, leading to AD pathogenesis.^222^ Adenosine triphosphate (ATP)-binding cassette transporter A1 (ABCA1) is known to regulate the stability of ApoE lipidation. Mutation in the ABCA1 gene has been reported to result accumulation of cholesterol in tissues, and AD pathogenesis.^222^ In contrast to PSEN1, PSEN2, and APP mutations, Clusterin (CLU) and Bridging Integrator 1 (BIN1) genes are risk factors for late-onset AD.^223−225^

According to CAS Content Collection, the largest number of documents associate APP, ApoE, PSEN-1, and CLU genes with AD pathogenesis (Figure 11B, lower panel). ABCA1, CLU, BIN1, and ApoE are the risk factors exhibiting the fastest growth in the number of documents in the last five years (2019–2023) (Figure 11B, upper panel).

We further considered the correlation between the genetic risk factors of the AD and the pharmacological targets. The co-occurrence of genetic factors and pharmacological targets in AD highlights the intricate interplay between genetics and potential treatments. Understanding this correlation can provide insights into the underlying mechanisms of the disease and guide drug development efforts. Likely intersections of genetic factors and pharmacological targets include: (i) precision medicine: genetic profiling can help tailor treatments based on individual risk factors, for example, patients with APOE ε4 might benefit more from certain amyloid-targeting therapies;^84,226^ (ii) APP and secretase inhibitors: understanding mutations in APP and presenilin genes has led to the development of secretase inhibitors to reduce amyloid-beta production;^218,227−229^ (iii) TREM2 and inflammation modulation: genetic studies linking TREM2 to AD have spurred interest in developing drugs that modulate microglial activity to reduce neuroinflammation;^230−232^ (iv) combination therapies: considering the multifactorial nature of AD, combination therapies targeting both amyloid-beta and tau, along with anti-inflammatory and neuroprotective strategies, may be more effective.^185,233,234^

Indeed, many pharmacological approaches for treating AD target the pathological hallmarks of the disease:

- Drugs targeting amyloid-beta include β-secretase inhibitors, γ-secretase inhibitors, and monoclonal antibodies that bind to amyloid-beta and promote its clearance.^235,236^
- Drugs targeting tau protein include tau aggregation inhibitors, tau kinase inhibitors, and immunotherapies aimed at reducing tau pathology.^237−240^
- Anti-inflammatory drugs target neuroinflammation, which is implicated in Alzheimer’s disease progression.^59,241,242^
- Neuroprotective agents aim to preserve neuronal function and viability, potentially slowing disease progression.^243−245^

**Figure 12:**
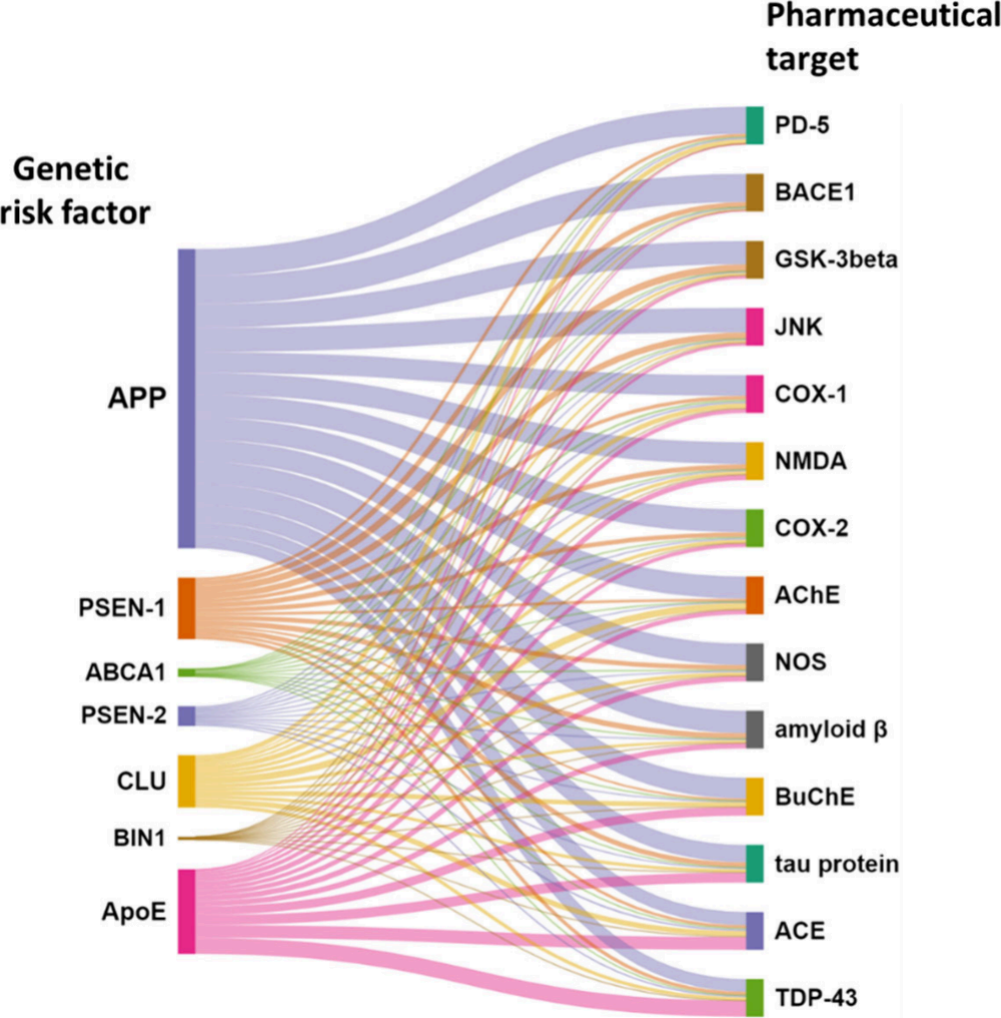
Co-occurrences of the genetic risk factors and pharmacological target proteins concepts in documents in the CAS Content Collection related to AD.

In effort to the get insight into the correlation between the genetic risk factors of AD and the pharmacological targets, we searched the documents of the CAS Content Collection related to AD for co-occurrences of certain major concepts related to the genetic risk factors and to the pharmacological targets within the AD-related document pool of the CAS Content Collection. The results are illustrated in Figure 12. APP co-occurs in documents with all target proteins, but with highest frequency with BACE1 and PD-4; ApoE most frequently co-occurs with TDP-43 and ACE, and PSEN-1–most frequently with GSK-3β and JNK.

- The correlation between APP and BACE1 is critical in the context of AD. APP is a transmembrane protein that, when cleaved by BACE1, produces beta-amyloid peptides. These peptides can aggregate to form amyloid plaques, a hallmark of AD. High levels of BACE1 activity can increase the production of Aβ, thus promoting plaque formation. Changes in the expression or activity of either APP or BACE1 can significantly affect Aβ production. Studies have shown that upregulation of BACE1 is observed in AD patients, which correlates with increased Aβ production. There is also evidence that oxidative stress and neuroinflammation can upregulate BACE1 expression and activity. Targeting BACE1 to reduce its activity is a potential therapeutic approach to lower Aβ production.^246−248^
- The correlation between APP and PD-4 in AD is an emerging area of research, albeit less explored than the correlation between APP and BACE1. Potential correlation include: (i) cAMP signaling: altered cAMP signaling has been implicated in neurodegenerative diseases, including AD.^249^ PD-4 role in cAMP degradation suggests that it could impact processes relevant to AD pathology;^250^ (ii) Neuroinflammation: elevated PD-4 activity can lead to reduced cAMP levels, potentially contributing to neuroinflammation.^251^ Neuroinflammation is a known factor in Alzheimer’s disease progression; (iii) Memory and cognitive function: PD-4 inhibitors have been studied for their potential to improve cognitive function by increasing cAMP levels, which could be beneficial in AD.^252,253^ Targeting PD-4 to modulate cAMP levels offers a potential therapeutic strategy. By inhibiting PD-4, it may be possible to reduce neuroinflammation and improve cognitive function in AD patients.^254^
- The correlation between ApoE and TDP-43 in AD is a significant topic as both proteins are implicated in neurodegenerative processes. It involves complex interactions between genetic risk factors, protein aggregation, and neuroinflammatory processes. ApoE4 appears to influence TDP-43 pathology, potentially exacerbating the overall disease pathology and cognitive decline.^203,255^ Further research is needed to fully understand the mechanisms underlying this interaction and to explore potential therapeutic strategies targeting both ApoE4 and TDP-43 in AD.
- The correlation between ApoE and ACE in AD involves their roles in neurodegenerative processes, vascular health, and inflammation. Both proteins have been implicated in the disease, with ApoE being a major genetic risk factor^84^ and ACE being involved in amyloid-beta metabolism and blood pressure regulation.^256^
- The correlation between PSEN-1 and GSK-3β in AD is significant due to their roles in amyloid-beta production and tau phosphorylation, respectively. Both proteins are implicated in key pathways that contribute to the pathogenesis of AD. PSEN-1 mutations lead to increased amyloid-beta, which can activate GSK-3β and exacerbate tau pathology.^257,258^
- The correlation between PSEN-1 and JNK in AD involves their roles in cellular signaling, amyloid-beta production, and neurodegeneration. Both PSEN-1 and JNK are implicated in pathways that contribute to AD pathology, particularly in relation to amyloid-beta accumulation, tau phosphorylation, and neuronal apoptosis.^28,246,257^ Understanding this interaction provides insights into the molecular mechanisms of AD and highlights potential therapeutic targets for managing both amyloid and tau-related aspects of the disease.

### Role of Primary Aging Hallmarks in Alzheimer’s Disease

Aging is characterized with a time-dependent gradual accumulation of cell damage and continual physiological functional decline. As such, it is also the most profound risk factor for many diseases. Neurodegenerative diseases, such as Alzheimer’s, Parkinson’s, and Huntington’s diseases, as well as sensorial disfunctions all increase considerably upon aging.^259−262^ With the growth in aging population and increasing burden of health care for people with age-related diseases, including AD, intense efforts have been put forth to understand and prevent the effects of aging. The major cellular and molecular hallmarks of aging have been identified and their relationships to age-related diseases, and especially to AD as one of the most common neurodegenerative disorders, have been explored.^263^

The hallmarks of aging include a variety of interrelated molecular and cellular mechanisms that act jointly to manage the aging process.^264^ Aging has been characterized as a progressive degeneration accompanied by processes like stem cell exhaustion, extracellular matrix modifications, cellular senescence, tissue inflammation, and metabolic dysfunction.^153,156^ These cellular and tissue modifications reflect inherent molecular deviations in mitochondria, epigenetics, DNA maintenance, proteostasis, intercellular interactions, and nutrient sensing, which cause genomic instability and impairment, including telomere dysfunction.^153,156^

DNA damage markers have been found in brain regions of AD patients, indicating that DNA damage may be an important pathological cause of AD, particularly in late-onset AD cases.^265−267^ Genomic instability impacts the expression of the genes linked to mitochondrial and metabolic dysfunction, altered proteostasis, and age-related inflammation (inflammaging), which are intrinsically involved in aging, and supports the view that DNA damage could be the root of aging and AD.^268,269^ Hence, targeting DNA damage or other aging hallmarks offers an approach to developing treatments to combat age-related diseases, including AD.^268^

Telomeres shorten with age, and some 50 nucleotides are lost upon each cell cycle. Neurons, as postmitotic cells, are not expected to have their telomeres shortened, yet they may still accumulate DNA damage, causing cellular senescence or even apoptosis.^270,271^ Neural stem cells, in contrast, are proliferative and affected by aging, so telomere maintenance is vital for their viability and self-renewal potential, while telomere shortening may trigger cognitive impairment.^272^ Epigenetic mechanisms are known to contribute directly to aging and aging-related diseases.^273^ It has been shown that epigenetics play a key role in maintaining genome integrity and regulating gene expression, and its dysfunction is closely related to AD pathogenesis.^274^

DNA methylation refers to the attachment of a methyl group to the DNA chain and is considered one of the aging hallmarks.^153^ Abnormal DNA methylation has been associated with many AD susceptibility genes, including amyloid precursor protein (APP), β- and γ-secretases, apolipoprotein E, to mention a few.^263,275,276^ As an organism ages, the proteome, like the genome, is easily damaged, so loss of proteostasis come up another hallmark of aging.^153^ At the early and advanced stages of AD, alteration of protein synthesis pathways, including nuclear chaperones, ribosomal proteins, and elongation factors, has been detected in the frontal cortex and hippocampus.^277,278^ Furthermore, proteins associated with various other biological processes are also aberrantly downregulated due to genomic instability in the AD brain.^279,280^

**Figure 13:**
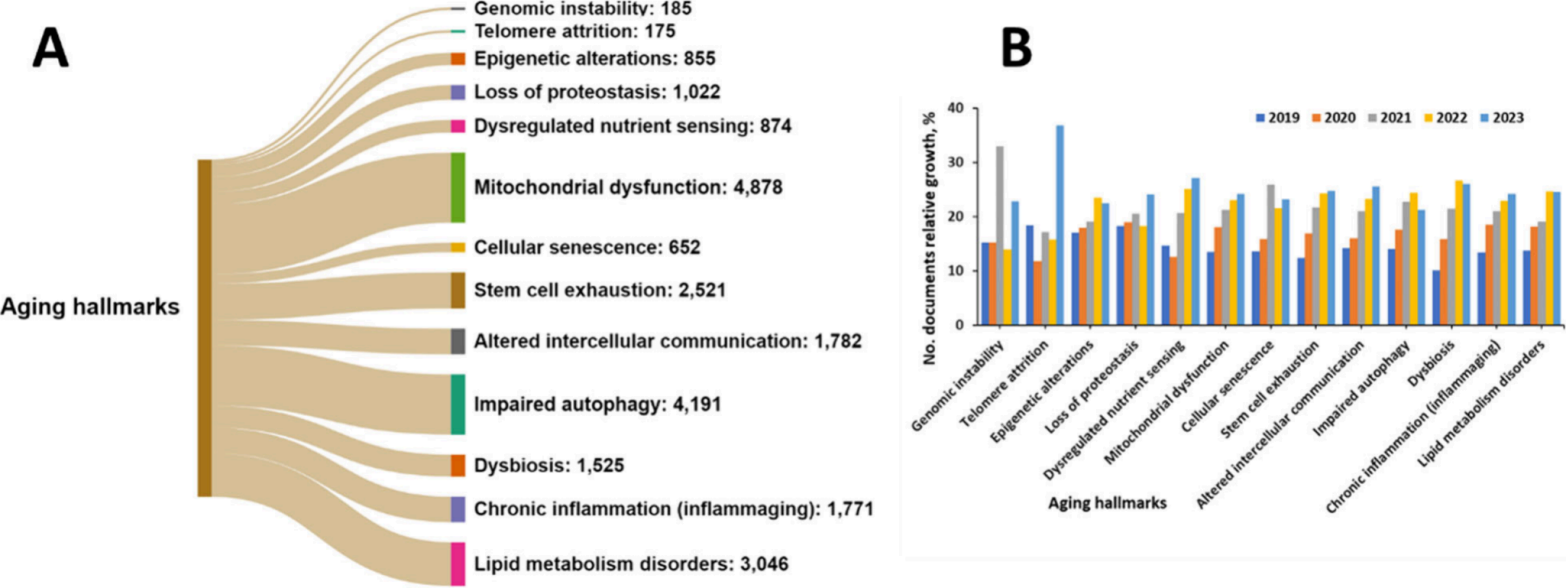
Correlation between primary aging hallmarks and AD as reflected by their co-occurrence in the documents in the CAS Content Collection: (A) number of documents and (B) relative growth in the years 2019–2023 related to the respective aging hallmark.

A search in the CAS Content Collection by SciFinder^17^ for co-occurrence of AD and the aging hallmarks term that AD is most often discussed in the context of mitochondrial dysfunction, impaired autophagy, and lipid metabolism disorders (Figure 13A). Indeed, mitochondrial dysfunction has been found related to virtually all the associated AD pathologies, including accumulation of plaques and tangles in the hippocampal and cortical neurons of the brain, abnormal microvasculature, interneuron miscommunication, enhanced β-amyloid production, elevated inflammatory response, advanced production of reactive oxygen species, impaired brain metabolism, tau hyperphosphorylation, and disruption of acetylcholine signaling.^281−289^

Autophagy is an evolutionarily conserved lysosome-dependent cellular pathway closely related to modulation of protein metabolism, by way of which damaged organelles and misfolded proteins are degraded and recycled to preserve protein homeostasis. Accumulating evidence has revealed that impaired autophagy critically contributes to AD pathogenesis.^290,291^ Immature autophagosome accumulation and dystrophic neurites have been detected in the AD patients brains.^291^ Furthermore, the expression of certain autophagy-related proteins has been reported to be downregulated in AD brains.^292−294^ Increasing evidence has recently indicated that dysfunctional autophagy is not just correlated with AD pathologies, but is possibly a causative factor for AD development. Various AD risk genes, including PSEN1, PICALM, CLU, TREM2 have been shown to modulate autophagy. Therefore, promoting autophagy to augment the elimination of misfolded proteins is recommended to be an opportunity for AD therapy.^290^

Though the abnormal accumulation of lipids has been described in the very first report of AD neuropathology,^295,296^ it has not been until recently that lipid homeostasis impairment has become a focus of AD research.^297^ Lipidomic and metabolomic studies have consistently exhibited alterations in the levels of various lipid classes emerging in early stages of AD brains. Multidimensional interactions between lipid metabolism and key AD pathogenic pathways including amyloidogenesis, bioenergetic deficit, oxidative stress, neuroinflammation, and myelin degeneration have been revealed.^297^

With respect to the relative growth in the number of documents correlating AD with various aging hallmarks, telomere attrition exhibits the highest growth, followed by the dysregulated nutrient sensing (Figure 13B). Indeed, the research of telomere biology is one of the most invested areas of research in AD prevention and treatment, due to its involvement in many age-related diseases.^298,299^ The presence of shorter telomeres in multiple somatic samples from AD patients, especially in leukocytes, has been reported.^300^ It has been suggested that the telomere shortening in peripheral leukocytes might be explained by certain known AD features on a molecular level such as high inflammatory cytokine levels and oxidative stress.^301−303^

Deregulated nutrient sensing is increasingly thought to play a role in the pathophysiology of neurodegenerative diseases such as AD.^304−306^ Nutrient sensing is increasingly emerging both as a key modulator of neurogenesis, and, through mTOR, of the autophagic process.^307,308^ The extensive role that deregulated nutrient sensing may play in dementia offers a possible therapeutic pathway, as many nutrient sensing-modulating therapeutics already exist.^309^

### Approved Drugs

Several prescription drugs have been approved by the US FDA for Alzheimer’s disease to help either manage the symptoms of or to treat the disease progression (Table 1), plus some in late phases of clinical trials (Table 2). The majority of the FDA-approved drugs work best for people in the early stages of Alzheimer’s disease. There are currently no known interventions that will cure Alzheimer’s disease.

**Table 2:**
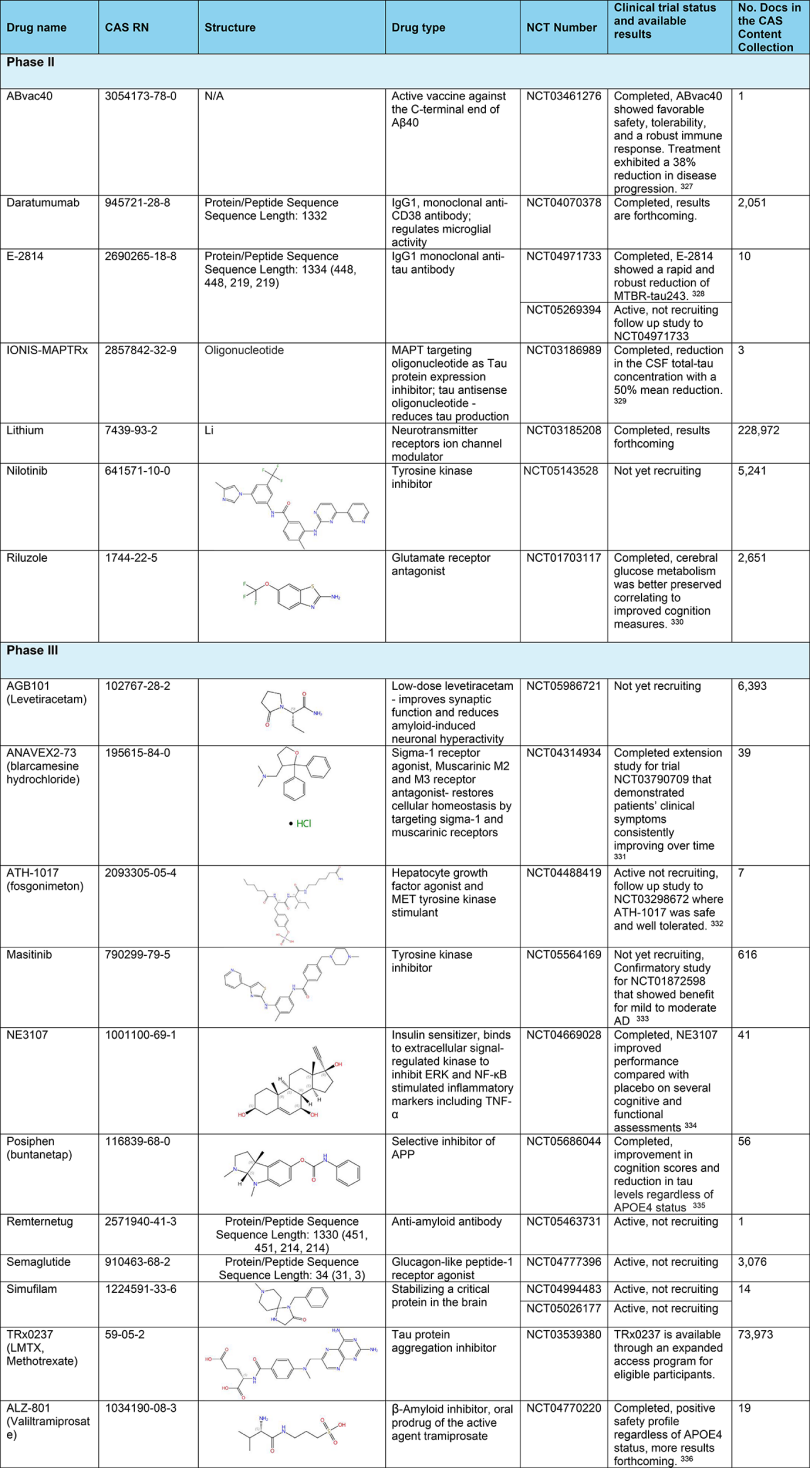
Exemplary Clinical Trials Phase II and III of Anti-Alzheimer Therapeutics^327−336^

**Cholinesterase inhibitors** are prescribed to treat symptoms associated with memory, thinking, language, judgment and other thinking processes. These medications decrease the breakdown of acetylcholine, a chemical messenger important for memory and learning. These drugs support communication between nerve cells.^120,124^

- Donepezil (Aricept) – approved to treat all stages of AD
- Rivastigmine (Exelon) – approved for mild-to-moderate AD
- Galantamine (Razadyne) – approved for mild-to-moderate AD
- Alpha-1062 (galantamine prodrug) – FDA New Drug Application acceptance for mild-to-moderate AD

**Glutamate regulators** are prescribed to improve memory, attention, thinking, language and the ability to perform simple tasks. This type of drug works regulate the activity of glutamate, a chemical messenger that helps the brain process information.^120,124,310^

- Memantine (Namenda) – approved for moderate-to-severe AD. It is an antagonist of the *N*-methyl-d-aspartate receptor (NMDA) subtype of glutamate receptor. By blocking the NMDA receptors, it helps regulate the activity of glutamate, preventing the overstimulation of these receptors and thereby protecting neurons from excitotoxicity.^125^

**Cholinesterase inhibitor + glutamate regulator** is a type of drug for combination therapy.^120,124^

- Donepezil + Memantine (Namzaric) – approved for moderate-to-severe AD.

**Orexin receptor antagonist** inhibits the activity of orexin, a type of neurotransmitter involved in the sleep–wake cycle.^120,124^

- Suvorexant (Belsomra) – approved for treatment of insomnia, effective for patients with mild to moderate AD.

**Atypical antipsychotics** are drugs that target the serotonin and dopamine chemical pathways in the brain. They are mainly used to treat schizophrenia and bipolar disorder and as add-on therapies for depressive disorders. These medications are also used to treat dementia-related behaviors.^120,124^

- Brexpiprazole (Rexulti) – approved for treatment of agitation associated with dementia due to AD.

**Figure 14:**
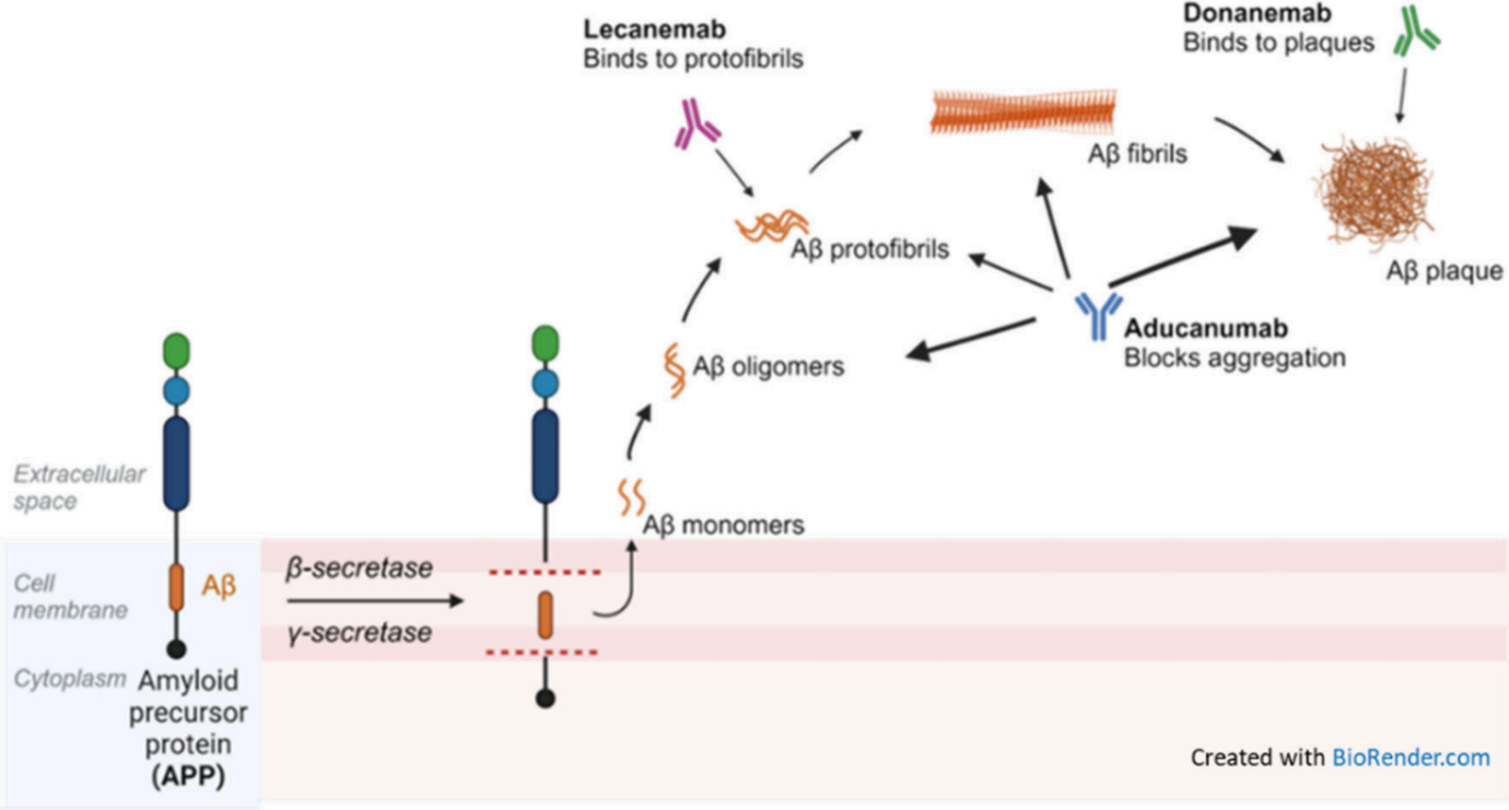
Scheme of the formation of amyloid plaques and target sites of the antiamyloid antibody drugs. Adapted from “Cleavage of Amyloid Precursor Protein” by BioRender.com (2024). Retrieved from https://app.biorender.com/biorender-templates.

**Antiamyloids** are the recent hope and promise in AD treatment.^142,311^ They work by attaching to and removing a protein that accumulates into plaques, β-amyloid, from the brain. These monoclonal antibody drugs target β-amyloid at a different stage of plaque formation (Figure 14). However, the results of the ongoing clinical trials are still controversial, and for the time being the benefits of these drugs seem to be harder to quantify than potential harms.^312,313^

- Aducanumab (Aduhelm) is an antiamyloid antibody, which received accelerated FDA approval for treatment of early AD in 2021.^314^ It has been the first medication to demonstrate that removing β-amyloid from the brain reduces cognitive and functional decline. Aducanumab is being discontinued by its manufacturer, Biogen. The company stated that this decision is not related to any safety or efficacy concerns, but rather to reallocating resources to other Alzheimer’s disease treatments.^315^ In fact, the approval of aducanumab was controversial due to mixed results in clinical trials regarding its efficacy in slowing cognitive decline, as well as regulatory and advisory criticisms, economic challenges, and limited market uptake.
- Lecanemab (Leqembi) is a humanized antiamyloid IgG1 monoclonal antibody that binds with high affinity to Aβ soluble protofibrils, received traditional FDA approval for the treatment of early AD.^316^ It is the second therapy to demonstrate that removing β-amyloid from the brain reduces cognitive and functional decline in patients living with early AD and the first to show clinical benefits.^317,318^ Latest data have shown that lecanemab reduces markers of amyloid in early Alzheimer’s disease and results in moderately lower decline on measures of cognition and function than placebo at 18 months.^319^ However, its use comes with notable risks and limitations, including side effects like Amyloid-Related Imaging Abnormalities (ARIA), high costs, and uncertain long-term efficacy. Longer trials are warranted to determine the efficacy and safety of lecanemab in early Alzheimer’s disease.^319^
- Donanemab (Kisunla) is an antiamyloid antibody, approved by the FDA for the treatment of early AD in patients with mild cognitive impairment or mild dementia.^320,321^ Application for full FDA approval initially took place in 2023 with the FDA granting final approval July 2024.^322^ It targets β-amyloid plaque, reducing the excess protein reducing cognitive and functional decline.^320^ As with lecanemab, donanemab has safety concerns including ARIA especially among patients who are ApoE ε4 homozygotes, high costs, and its limitation to the treatment of patients with early disease activity. Continued clinical trials are needed to assess and monitor both safety and efficacy.

### Drugs in Clinical Trials

**Figure 15:**
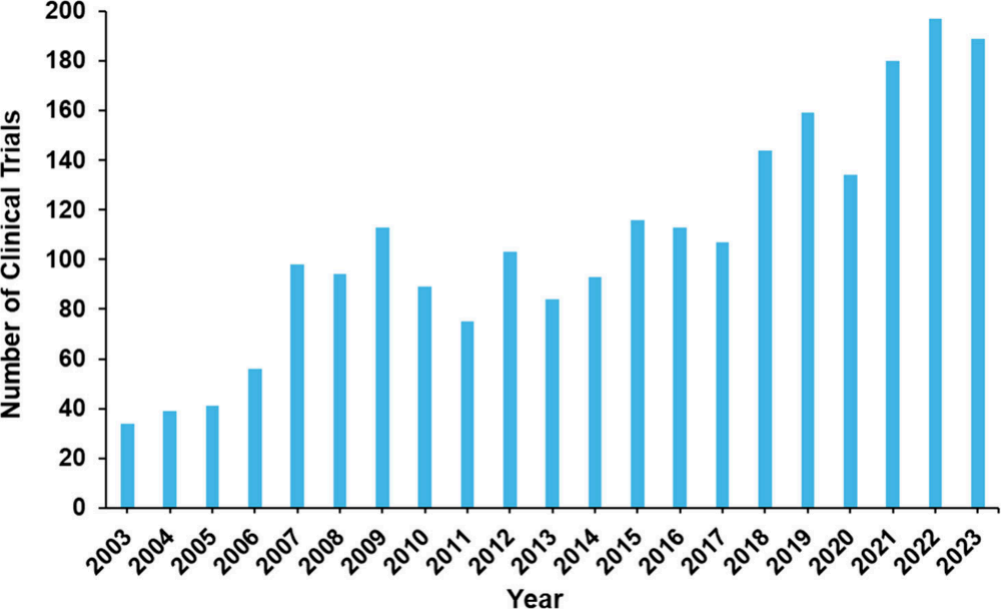
Number of Alzheimer’s disease therapeutic clinical trials by year.

Clinical trials researching the treatment of Alzheimer’s disease are explored in this section to gain an overall view of the past and current state of clinical development. Over 2200 clinical trials have been registered on clinicaltrials.gov over the last 20 years for Alzheimer’s diseases, reinforcing a strong interest in clinical development of treatments for this devastating disease. Figure 15. shows an increasing oscillating curve starting at around 40 clinical trials and raising to around 200 clinical trials per year, between the years 2003 to 2023.

**Figure 16:**
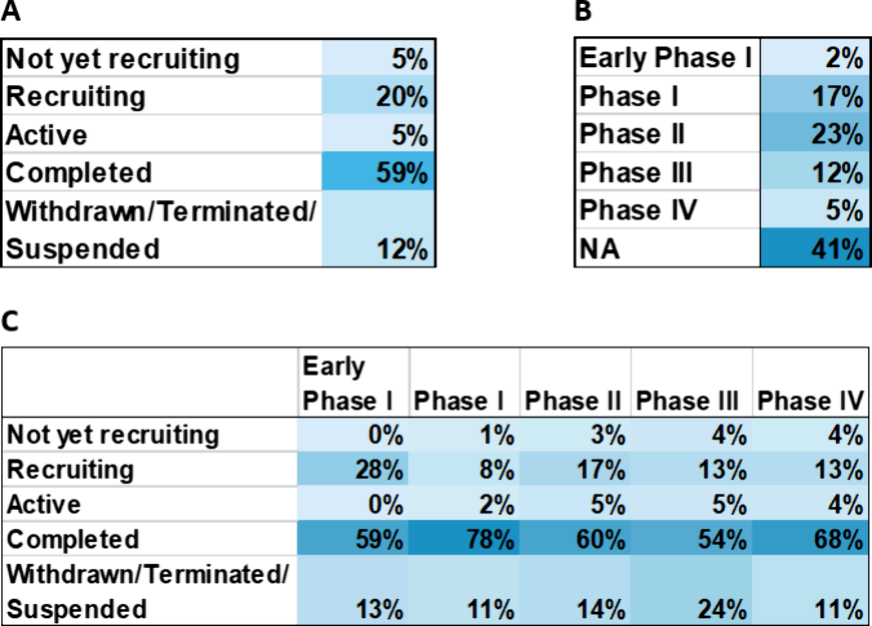
Percentage of therapeutic Alzheimer’s disease clinical trials for various phases and statuses: (A) overall clinical trial phase development, (B) overall clinical trial statuses, (C) clinical trial phases compared against statuses. Phase I/II and Phase II/III studies are classified under Phase II and Phase III studies, respectively.

Analysis of Alzheimer’s disease therapeutic clinical trials reveals that around 41% of all trials are not phased (Figure 16A), including trials such as ones testing medical devices or behavioral interventions. The phase that contains the next largest group of trials is Phase I and Phase II studies, researching the safety and efficacy of newer anti-Alzheimer’s disease agents. Over half of all clinical trials in the past 10 years have been completed (Figure 16B). The status with the next largest group of trials is the recruiting status which is encouraging as new clinical trials are created and carried out to research the treatment of Alzheimer’s disease, offering hope to patients worldwide.

Exemplary Phase II and Phase III clinical trials and their studied drugs are highlighted in in Table 2 along with their CAS RN, structure, drug type, and number of publications in the CAS Content Collection.

### Drug Repurposing

Since there is no cure for AD, drug repurposing studies have been intensely searching to identify existing drugs that could be repositioned to treat AD.^337−342^ As pharmaceutical development process is both time-consuming and costly, drug repurposing provides a chance to accelerate it by exploring the AD-related effects of agents approved for other disorders. These drugs have established safety profiles, pharmacokinetic description, formulations, dosages, and manufacturing procedures. Recently, *in silico* pharmacology has been widely applied and various computer applications including machine learning and artificial intelligence approaches have been explored in identifying potential drugs for repurposing to AD.^343−346^ Drug repurposing has already been attempted in AD with various methodologies applied and several clinical trials are currently evaluating drug-repurposing candidates for AD.^337,347−349^ Fifty widely discussed drugs for AD repurposing^337−349^ are summarized in Table 3.

**Table 3:** Exemplary Drugs Commonly Considered for Repurposing to AD, along with the Number of Documents in the CAS Content Collection Related to Them

drug	CAS RN	no. docs	drug type
Lithium	7439-93-2	958	psychiatric
Metformin	657-24-9	741	antidiabetic
Risperidone	106266-06-2	704	antipsychotic
Nicotinamide	98-92-0	693	vitamin
Atorvastatin	134523-00-5	574	cardiovascular
Tamoxifen	10540-29-1	503	anticancer
Clozapine	5786-21-0	437	antipsychotic
Pioglitazone	111025-46-8	436	antidiabetic
Tacrolimus	104987-11-3	416	immunologic
Aripiprazole	129722-12-9	332	antipsychotic
Dronabinol	1972-08-3	326	appetite stimulant, antiemetic
Riluzole	1744-22-5	306	neurologic
Verapamil	52-53-9	305	Ca-channel blocker, antihypertensive
Venlafaxine	93413-69-5	291	psychiatric
Escitalopram	128196-01-0	268	psychiatric
Bromocriptine	25614-03-3	260	dopamine agonist
Bupropion	34911-55-2	257	antidepressant
Liraglutide	204656-20-2	253	antidiabetic
Levetiracetam	102767-28-2	246	Neurologic, antiepileptic
Methylphenidate	113-45-1	231	psychiatric
Mirtazapine	85650-52-8	230	psychiatric
Raloxifene	84449-90-1	229	estrogen receptor modulator
Thalidomide	50-35-1	225	immunomodulator
Vorinostat	149647-78-9	215	anticancer
Losartan	114798-26-4	188	cardiovascular
Leuprolide	53714-56-0	188	hormonal
Sunitinib	557795-19-4	181	anticancer
Lenalidomide	191732-72-6	176	anticancer, hematologic
Nilotinib	641571-10-0	176	anticancer
Amlodipine	88150-42-9	173	cardiovascular
Brexanolone	516-54-1	169	psychiatric
Zidovudine	30516-87-1	159	HIV antiviral
Telmisartan	144701-48-4	150	cardiovascular
Zolpidem	82626-48-0	148	neurologic
Prazosin	19216-56-9	133	cardiovascular
Cilostazol	73963-72-1	113	hematologic
Candesartan	139481-59-7	108	cardiovascular
Allopurinol	315-30-0	105	uric acid reducer
Deferiprone	30652-11-0	100	hematologic
Salsalate	552-94-3	98	anti-inflammatory
Montelukast	158966-92-8	95	anti-inflammatory
Vandetanib	443913-73-3	92	anticancer
Efavirenz	154598-52-4	92	HIV antiviral
Perindopril	82834-16-0	87	cardiovascular
Zopiclone	43200-80-2	78	neurologic
Valacyclovir	124832-26-4	76	antiviral
Dabigatran	211914-51-1	69	hematologic
Sodium phenylbutyrate	1716-12-7	61	anticancer, cystic fibrosis
Dapagliflozin	461432-26-8	60	antidiabetic

**Figure 17:**
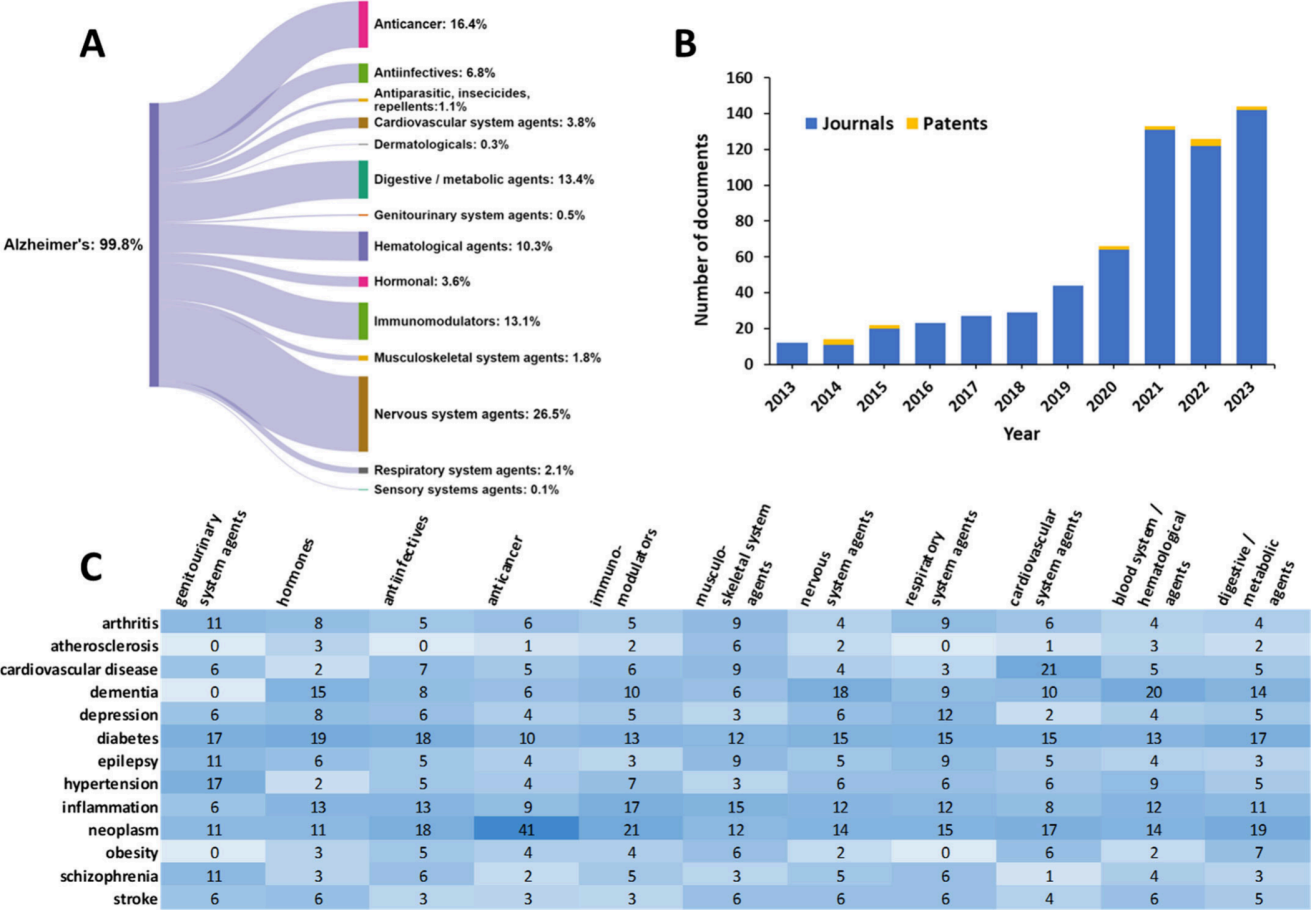
(A) Classes of drugs explored for repurposing to AD treatment with associated number of documents indicated. (B) Annual growth of the drug repurposing-related documents (journal articles and patents). (C) Correlation between diseases co-occurring with AD and potential drugs for repurposing to AD as judged by the concepts co-occurrence in the CAS Content Collection documents.

We identified over 700 documents in CAS Content Collection discussing drug repurposing for AD. The number of AD drug repurposing publications increased dramatically over the past decade–from 13 documents in 2013 to 147 in 2023 (Figure 17B). The distribution of the considered potential repurposed drug classes with respect to their original disease targets according to the CAS Content Collection is illustrated in Figure 17A. Predictably, drugs for repurposing to AD are most often searched within the pool of nervous system agents. Anticancer drugs, metabolic agents, and immunomodulators are also between the hopeful drugs for repurposing to AD (Figure 17A).

We further examined the correlation between diseases that co-occur with AD and the exploration of drug repurposing. The correlation between the concepts related to diseases co-occurring with AD and the exploring classes of drugs for repurposing to AD is illustrated in Figure 17C. In addition to certain obvious correlations such as cancers (neoplasm) – anticancer agents and cardiovascular diseases–cardiovascular system agents, there are some worth mentioning:

#### Dementia—Hematological agents

Studies have implicated vascular disorders as risk factors for dementia. Biological and epidemiological evidence have been reported concerning the role of certain hematologic factors such as homocysteine, cholesterol, fatty acids, antioxidants, and C-reactive protein in dementia.^350^ Abnormal (low or high) levels of hemoglobin have been associated with an enhanced risk of dementia, including AD, which may be associated with differences in white matter integrity.^351^ Association has been reported between blood leukocyte counts and increased risk of AD.^352^

#### Diabetes—Hormones

An imbalance in sex hormones has been identified as having a critical impact on type 2 diabetes. Androgens have a noteworthy sex-dimorphic association with type 2 diabetes, since hyperandrogenism in females and hypogonadism in males are risk factors for type 2 diabetes. Thus, treatments aimed at correcting these sex hormone imbalance may prevent the development of type 2 diabetes or help in its treatment.^353^

#### Inflammation—Immunomodulators

Inflammation is a central part of autoimmune diseases, which are caused by dysregulation of the immune system, involving imbalance between pro-inflammatory vs anti-inflammatory mediators.^354^

#### Neoplasm—Immunomodulators

Immunotherapy is treatment that uses your body’s own immune system to help fight cancer. Many immunotherapeutic agents operate by activating an efficient antitumor response or reversing tumor-mediated immunosuppression through modulation of significant regulatory pathways.^355^

Since AD is a complex neurodegenerative disorder, individuals with AD often have other health conditions or comorbidities. Some of these comorbidities may share underlying biological pathways or mechanisms with AD. By examining diseases that commonly co-occur with Alzheimer’s, researchers may identify potential candidate drugs for repurposing. Overall, understanding the relationship between comorbidities of AD and the potential for drug repurposing can provide valuable insights into the development of new treatments or therapeutic strategies for this devastating condition. Exploration of drug repurposing for AD involves leveraging the shared pathophysiological mechanisms and existing drug libraries of comorbid conditions, employing a combination of biological and computational approaches, and conducting rigorous clinical trials to validate the efficacy and safety of repurposed drugs.

## Future Directions of Research

Research on Alzheimer’s disease is a dynamic field, with ongoing efforts aimed at understanding the underlying causes of the disease, developing effective treatments, and ultimately finding a cure. Understanding the molecular and cellular mechanisms underlying AD, including the role of β-amyloid plaques, tau protein tangles, neuroinflammation, and synaptic dysfunction, is a major focus of research. Advances in techniques such as imaging, genetics, and molecular biology are providing insights into the early stages of AD and potential targets for intervention.

Identifying reliable biomarkers for AD, including blood-based markers, cerebrospinal fluid markers, and imaging biomarkers, is crucial for early detection, accurate diagnosis, and monitoring disease progression. Recently, the first Alzheimer’s treatments targeting disease pathophysiology became available to patients and accelerated this need. Research is ongoing to develop new and more sensitive biomarkers that can detect AD pathology in its earliest stages, even before symptoms appear, when treatments are most effective.^113,356^ Noninvasive diagnostic tests are showing great promise in detecting β-amyloid and tau protein biomarkers equivalent or superior to FDA-approved CSF tests in the detection of AD pathology.^111^ These diagnostic assays, more easily accessible, will help alleviate the bottleneck and unmet needs of healthcare professionals and patients in evaluating them for AD.

Drug development efforts are focused on targeting various aspects of AD pathology, including reducing β-amyloid production or aggregation, inhibiting tau protein accumulation, modulating neuroinflammation, and promoting synaptic function and neuronal survival. Immunotherapy approaches, such as monoclonal antibodies targeting β-amyloid or tau proteins, are being investigated in clinical trials as potential disease-modifying treatments. Combination therapies targeting multiple pathways involved in AD pathogenesis are also being explored.

Advances in genetics and personalized medicine are leading to a better understanding of individual differences in AD risk, progression, and response to treatment. Precision medicine approaches aim to tailor treatments and interventions to the specific genetic and biological characteristics of each individual, potentially improving treatment efficacy and minimizing side effects.

Research continues to explore the efficacy of nonpharmacological interventions, such as cognitive training, physical exercise, diet, and lifestyle modifications, in reducing the risk of AD and improving cognitive function and overall brain health. Multimodal interventions combining various lifestyle factors, such as diet, exercise, cognitive stimulation, and social engagement, are being studied for their potential synergistic effects on brain health.

The analysis of large-scale data sets, including genetic data, clinical data, imaging data, and digital biomarkers, using advanced computational techniques such as machine learning and artificial intelligence, holds promise for uncovering new insights into AD and predicting disease progression. Collaborative initiatives such as the AD Neuroimaging Initiative (ADNI)^357^ and the Global Alzheimer’s Association Interactive Network (GAAIN)^358^ facilitate data sharing and collaboration among researchers worldwide.

While significant challenges remain in the fight against AD, sustained efforts in research, advocacy, and care have the potential to improve outcomes for individuals affected by the disease and advance our collective efforts toward a future without Alzheimer’s.
